# Haplotype‐Resolved 3D Genomic Landscapes and Their Impacts on Agronomic Traits in Grapevine

**DOI:** 10.1002/advs.202521838

**Published:** 2026-04-15

**Authors:** Yanling Peng, Lianzhu Zhou, Xinyue Fang, Ruoyan Zhao, Qi Xu, Mengrui Du, Ting Hou, Guizhou Huang, Yingchun Zhang, Sheng Yan, Ruo Yang, Zhongxin Jin, Yang Dong, Yanshuai Xu, Hua Xiao, Xiaodong Xu, Yi Liao, Xiping Wang, Yongfeng Zhou

**Affiliations:** ^1^ State Key Laboratory For Crop Stress Resistance and High‐Efficiency Production College of Horticulture Northwest A&F University Yangling Shaanxi China; ^2^ State Key Laboratory of Tropical Crop Breeding Shenzhen Branch Guangdong Laboratory of Lingnan Modern Agriculture Key Laboratory of Synthetic Biology Ministry of Agriculture and Rural Affairs Agricultural Genomics Institute At Shenzhen Chinese Academy of Agricultural Sciences Shenzhen China; ^3^ State Key Laboratory of Tropical Crop Breeding Tropical Crops Genetic Resources Institute Chinese Academy of Tropical Agricultural Sciences Haikou China; ^4^ College of Horticulture South China Agricultural University Guangzhou China

**Keywords:** 3D genomes, berry color, clonal crops, grapevine, haplotype‐resolved, seedlessness, structural variations

## Abstract

In clonally propagated crops, extensive divergence between haplotypes complicates transcriptional regulation. However, the contribution of the three dimensional (3D) genome organization to these allelic differences and agronomic traits remains unclear. Here, we generated haplotype‐resolved 3D genome landscapes for grapevine cultivars with contrasting berry colors and seed traits, integrating them with genomic, epigenomic, and transcriptomic profiles. We found profound 3D architectural divergence between haplotypes, spanning from large‐scale A/B compartments down to a level of topologically associated domain (TAD) boundary variation (18.53%∼23.01%) that approached inter‐cultivar differences (18.74~21.62%) (*p* > 0.05). A key mechanism driving these effects involves large‐scale, haplotype‐specific transitions between distinct TAD states (Active, Inactive, Heterochromatic), which asymmetrically regulate transcription and alter local DNA methylation patterns. Importantly, these structural rearrangements, including TAD boundary shifts, are strongly associated with underlying structural variants (SVs). Critically, this regulatory cascade impacts key agronomic loci, genes controlling berry color (e.g. *VvMYBA*) and seedlessness determination (e.g. *VvSUS2*) were positioned at cultivar‐specific TAD boundaries, exhibiting presence/absence variations and differential expression patterns. Our findings support a mechanistic model wherein phased 3D chromatin architecture and heterozygous SVs are strongly associated with the regulation of key agronomic traits, paving the way for accelerating the genetic improvement of clonal crops.

## Graphical abstract

This study presents a haplotype‐resolved 3D genomic landscape of grapevine, revealing that structural variations (SVs) are closely associated with phased topologically associating domain (TAD) boundary transitions. These rearrangements coordinate with allele‐specific DNA methylation (ASM) and allele‐biased gene expression (ASE) to shape key agronomic traits such as berry color and seedlessness, facilitating precision crop breeding.

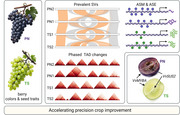

## 1 Introduction

Clonally propagated crops, such as grapevine, accumulate structural variations (SVs) in their genomes over successive generations of asexual reproduction due to the absence of genetic recombination and mutation‐purging mechanisms [1, 2, 3, 4, 5, 6]. The accumulation of SVs in clonal crops results in highly heterozygous genomes where the two parental haplotypes can differ significantly in gene content and regulatory architecture [1, 7]. Indeed, our previous work established that this high SV burden gives rise to a significant number of hemizygous genes, genes present on only one of two homologous chromosomes, which are in turn subject to complex gene regulation, exhibiting altered expression levels and distinct epigenetic patterns that influence traits like fruit development [7]. The broader functional consequences of such haplotype‐specific expression are profound, affecting key agronomic traits, such as sex determination [1, 8, 9], the berry color [1, 10] and seed development [11]. However, while the link between clonal propagation, SV accumulation, and altered gene expression is clear, the precise high‐level regulatory mechanisms that translate these structural differences between haplotypes into functional, trait‐defining variation remain largely unknown. This knowledge gap presents a major hurdle for understanding genome function and accelerating genetic improvement in clonal crops.

A compelling mechanism to explain this connection lies in the three‐dimensional (3D) architecture of the genome. The folding of chromatin into a hierarchy of functional domains, including chromosomal compartments (A/B compartments), topologically associating domains (TADs), and chromatin loops, is a critical layer of gene regulation that orchestrates enhancer‐promoter communication [12, 13, 14, 15]. In plants, studies using Hi‐C technology have begun to unravel these complex landscapes. For instance, investigations in polyploid cotton have revealed differences in the spatial organization between subgenomes, where the switching of chromatin compartments from active to inactive states is strongly associated with gene silencing during fiber development [16, 17]. Furthermore, comparative analyses in cotton have demonstrated that subgenomes exhibit distinct 3D reorganization dynamics across different tissues [18].

Crucially, this 3D architecture can be directly reconfigured by SVs, which can disrupt TAD boundaries, merge domains, or create novel chromatin contacts that lead to gene misexpression [19, 20, 21, 22, 23, 24]. The functional importance of such remodeling is compelling. SVs ranging from TE insertions to chromosomal rearrangements can reshape TADs, altering fiber development in cotton [23, 25] and diversifying wing patterns in butterflies [24]. Despite this progress, the impact of SVs on 3D genome architecture has remained unexplored at a phased, haplotype‐resolved level in highly heterozygous clonal crops like grapevine.

The central hypothesis of this study is that the extensive SVs differentiating the two haplotypes in a grapevine cultivar shape the formation of distinct, haplotype‐specific 3D genome architectures. We posit that these architectural differences, arising from SVs that alter regulatory domains [20, 21], in turn modulate the local epigenetic landscape and transcriptional activity, providing a mechanistic basis for the allele‐biased expression of genes controlling key agronomic traits [1, 11, 26].

Therefore, this study moves beyond a linear genome view to investigate how the two distinct parental haplotypes within highly heterozygous grapevine cultivars, ‘Pinot Noir’ (PN) and ‘Thompson Seedless’ (TS), are organized in three‐dimensional space and how this organization impacts function. We were driven by a series of central questions. First, we asked how significantly the 3D chromatin architecture differs between haplotypes within an individual and whether this divergence is as pronounced as that between different cultivars. We then investigated the underlying molecular mechanisms, questioning how these haplotype‐specific architectures correlate with epigenetic marks and lead to allele‐biased gene expression. Crucially, we sought to investigate whether SVs are strongly associated with this 3D remodeling. Finally, we addressed the ultimate agronomic significance by asking if these structural differences support a mechanistic model for key traits like berry color and seedlessness. By integrating Hi‐C with multi‐omic datasets to address these questions, our study aims to establish a direct link between haplotype‐resolved genome structure and trait regulation, offering a new framework for the genetic improvement of clonal crops.

## 2 Results

### 2.1 Haplotype‐Resolved A/B Compartments Landscapes in TS and PN

**FIGURE 1:**
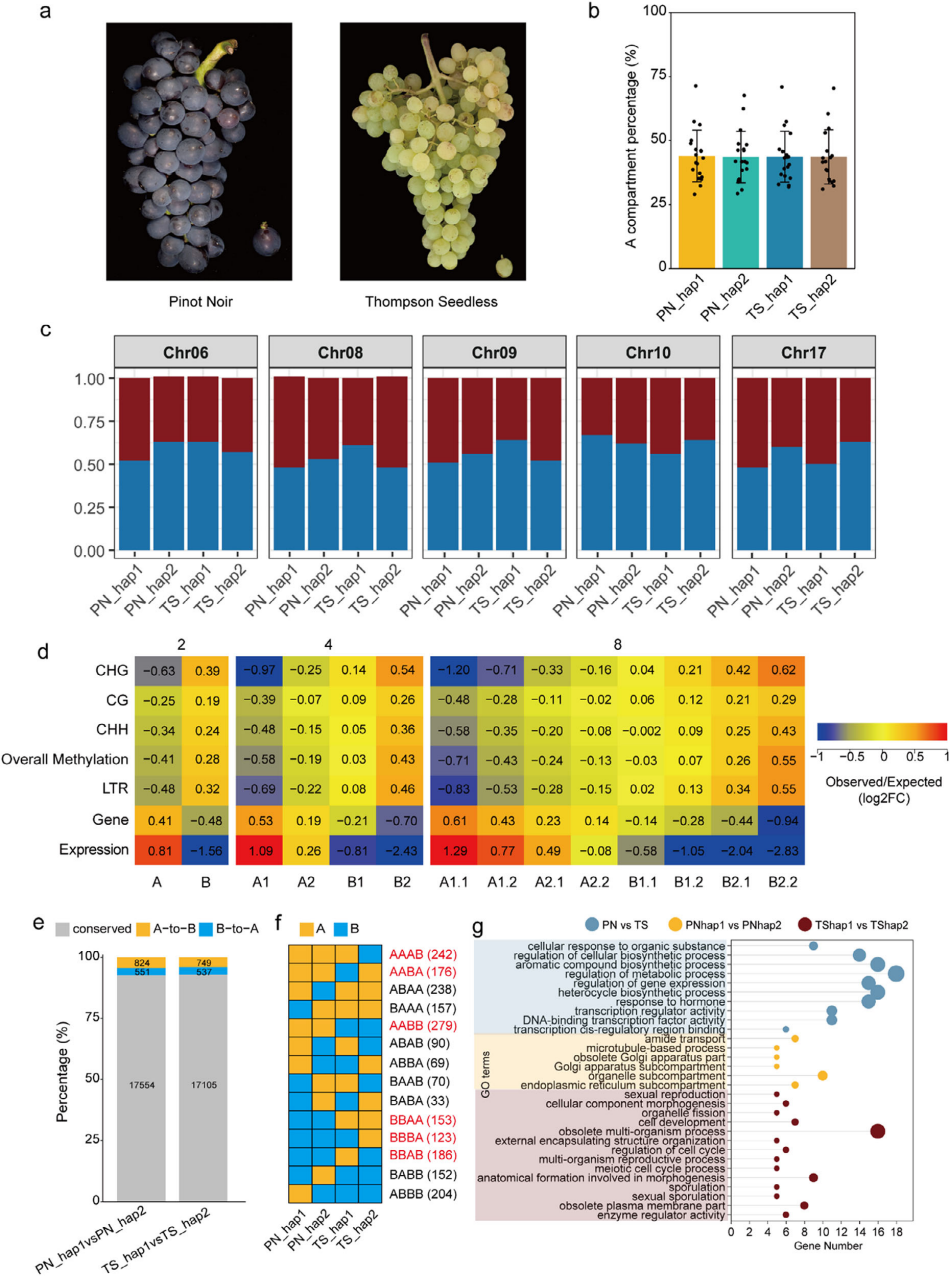
Haplotype‐resolved A/B compartments in TS and PN. (a) Morphological traits of PN and TS. (b) Proportion of ‘A’ compartments per chromosome in haplotype‐resolved genomes. Each point represents a chromosome; data are shown as mean ± standard deviation (s.d.). (c) A/B compartment proportions on chromosomes 06, 08, 09, 10, and 17 in four haplotype genomes. (d) Enrichment of genomic/epigenomic features (rows) across subregional compartments (columns) in TS_hap1. Log2‐fold changes between observed and expected medians are color‐coded. (e) Proportion of conserved genes and genes undergoing A/B compartment change between PN_hap1‐PN_hap2 and TS_hap1‐TS_hap2. (f) Number of homologous genes with A/B compartment changes across four haplotype genomes. Yellow: genes in ‘A’ compartment; blue: genes in ‘B’ compartment. (g) GO enrichment analysis of homologous genes undergoing compartment change and differential expression in three comparations.

To resolve the 3D genome architecture at the haplotype level, we constructed phased Hi‐C maps for two grapevine cultivars, PN and TS (Figure 1a). The high consistency between two independent processing pipelines (HiCExplorer and HiC‐Pro) confirmed the robustness of our analyses (Figure S1; Tables S1 and S2; Dataset S1). At the megabase scale, all four haplotype genomes (PN‐hap1/2, TS‐hap1/2) displayed clear checkerboard patterns indicative of A/B compartments, with active ‘A’ compartments generally corresponding to gene‐rich chromosome arms and inactive ‘B’ compartments aligning with gene‐poor pericentromeric regions (Figures S2 and S4a–c).

While the overall proportion of A/B compartments was stable across haplotypes (Figure 1b; Figure S3a), we observed striking examples of localized, haplotype‐specific compartmentalization. For instance, chromosomes 06, 08, 09, 10 and 17 in PN and TS showed clear differences in ‘A’ and ‘B’ compartment proportions between haplotypes 1 and 2 (Figure 1c). Similar patterns of proportion differences in A/B compartment proportions were observed on other chromosomes, demonstrating large‐scale chromatin state switching within a single plant (Figure S3b).

We first examined the relationship between grapevine A/B compartments and genomic as well as epigenomic features. ‘A’ compartments were predominantly located in chromosome arms, whereas ‘B’ compartments were concentrated near centromeres, mirroring the overall distribution of genes and TEs (Figure S4a–c). Iterative partitioning revealed nested subcompartments, averaging ∼600 kb in length, whose ranks correlated strongly with genomic and epigenomic profiles (Figure 1d; Figure S4d–f). A‐type subcompartments were enriched in genes and exhibited higher expression, while B‐type subcompartments were TE‐rich and hypermethylated in CG, CHG, and CHH contexts (Figure 1d; Figure S4e,f). These multiscale subcompartments likely reflect fine‐scale epigenetic variation shaping chromatin organization, and their features were largely consistent between haplotypes.

We then quantified A/B compartmentalization changes and their effects on homologous genes. Between PN_hap1 and PN_hap2, 7.3% (1375) of homologous genes shifted compartment status, including 4.4% (824) with an ‘A’ to ‘B’ change and 2.9% (551) with a ‘B’ to ‘A’ change (Figure 1e). TS_hap1 vs. TS_hap2 showed similar proportions (7.0%: 4.1% ‘A’ to ‘B’, 2.9% ‘B’ to ‘A’; Figure 1e). Between cultivars, 10.1% (1910) of homologous genes differed, but only 1.5% (279; AA to BB) and 0.8% (153; BB to AA) changed in both haplotypes, with the remainder (7.8%) altering in a single haplotype (Figure 1f). Notably, the proportion of differentially expressed homologous genes was significantly higher in regions undergoing compartment shifts than in stable regions (Figure S5a), indicating that A/B compartment transitions can promote allele‐specific expression. Genes undergoing A/B compartment change within different haplotypes of the same cultivar and between cultivars are enriched in different functional categories (Figure 1g; Figure S5b,c). Among them, genes undergoing A/B compartment change and differentially expressed in TS are significantly enriched in biological processes related to seed development, such as ‘sexual reproduction’ and ‘sexual spore formation’ (Figure 1g).

### 2.2 Haplotype‐Resolved TADs in TS and PN

**FIGURE 2:**
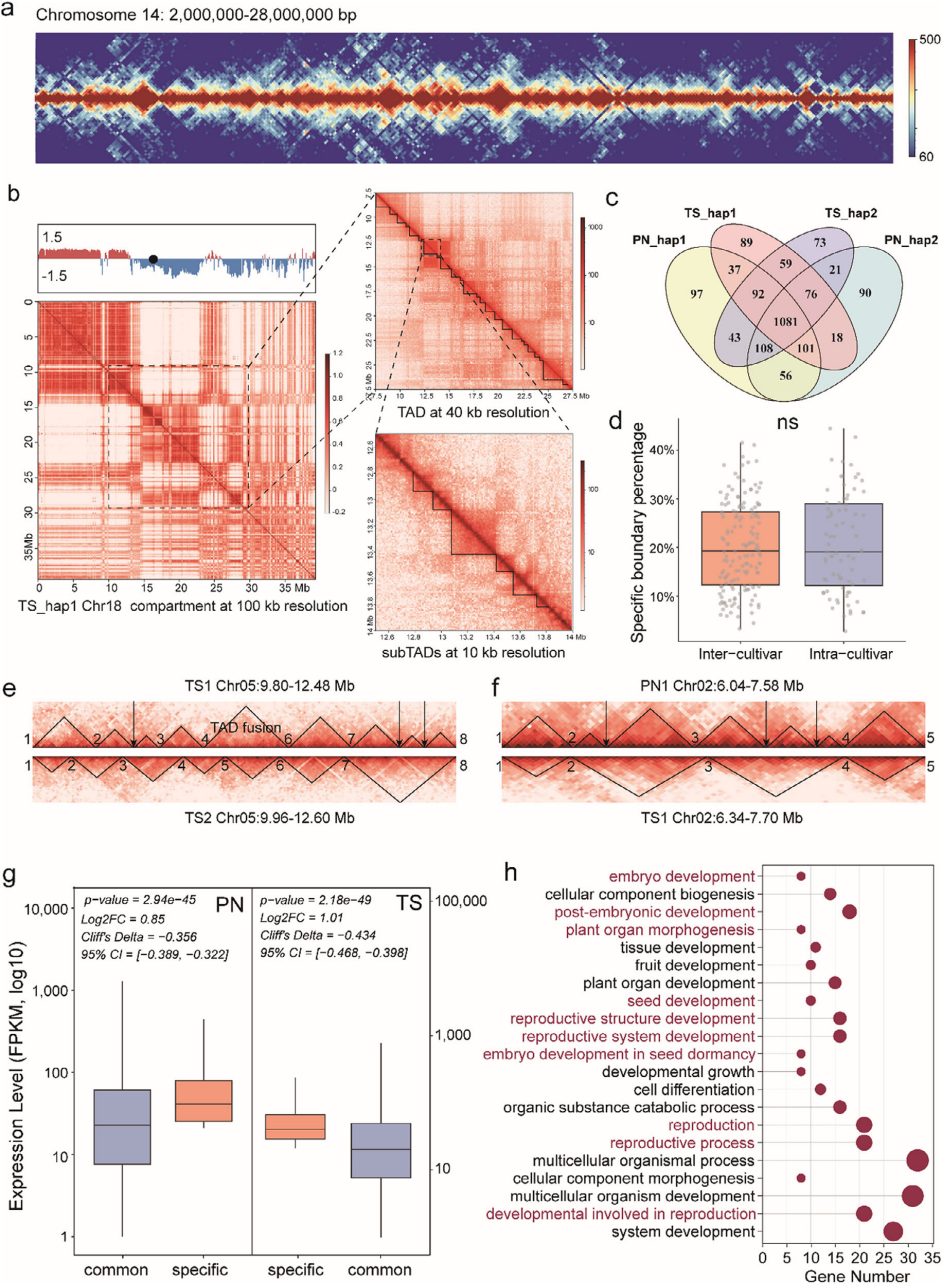
Haplotype‐resolved TAD in TS and PN. (a) Example of TAD‐like domains in the 26 Mb region of Chromosome 14. The Hi‐C interaction map of TS_hap1 at 100 kb resolution shows TADs as squares or rectangles with high contact frequencies. (b) 3D chromatin structure of the TS_hap1 genome. Left: Hi‐C interaction map and E1 values of Chromosome 18 (Chr18), showing chromatin compartments. Top right: zoomed‐in 100 kb map at 40 kb resolution, highlighting TADs (black lines). Bottom right: further zoomed 40 kb map at 10 kb resolution, revealing subTADs (black lines). (c) Number of specific and conserved TAD boundaries across haplotype‐resolved genome, using PN_hap1 as the reference. (d) Percentage of specific TAD boundaries in inter‐ and intra‐cultivar comparisons, evaluated across all 19 individual chromosomes. (e) TAD structure in the co‐linear region of Chr05 between TS_hap1 and TS_hap2 and, (f) in the co‐linear block of Chr02 between PN_hap1 and TS_hap1. Black triangles mark TADs, red highlights strong interactions, numbers indicate conserved TAD boundaries, and black arrows mark specific TAD boundaries. (g) Comparison of gene expression levels at common vs. cultivar‐specific TAD boundaries at 30 kb bins. The *y*‐axis represents actual FPKM values plotted on a logarithmic (log10) scale and the magnitude of the expression differences is explicitly quantified and annotated using Log2 Fold Change (Log2FC) and Cliff's Delta with 95% confidence intervals (CI). (h) GO enrichment analysis of differential expression genes at TS‐specific TAD boundaries. The boxes in (d and g) indicate the interquartile range (IQR) with the median denoted by the horizontal center line, and whiskers extend to 1.5 times the IQR. Statistical significance was evaluated using a two‐sided Wilcoxon rank‐sum test (ns: not significant, *p*‐value > 0.05).

To investigate chromatin organization at a higher resolution, we identified TADs across the phased genomes. Each haplotype contained approximately 1500~1600 TADs, with a consistent average size of ∼300 kb (Figure 2a; Figure S6a). These TADs were organized hierarchically within the larger A/B compartments, indicating a multi‐level chromatin structure (Figure 2b).

Our comparative analysis revealed a dynamic TAD landscape. Within a single cultivar, the proportion of haplotype‐specific TAD boundaries was remarkably high (95% confidence intervals [CI]: 18.5%~23.0%). Strikingly, this profound intra‐cultivar divergence closely approached and heavily overlapped with the variation observed in comparisons between distinct cultivars (95% CI: 18.7%~21.6%) at the baseline 30 kb analytical resolution (Figure 2c,d). To quantitatively substantiate this finding and address potential methodological biases, we assessed the statistical robustness of these variations by performing a stringent sensitivity analysis, varying the boundary‐matching tolerance from 20 to 50 kb. Across all tested tolerances, bootstrap analyses revealed that the 95% CIs for intra‐cultivar variation consistently overlapped with those of inter‐cultivar differences (e.g., at 50 kb: Intra 14.89%~21.19% vs. Inter 15.63%~19.24%; at 20 kb: Intra 20.68%~24.26% vs. Inter 21.92%~24.40%). Global Wilcoxon rank‐sum tests confirmed no significant difference between the two groups, regardless of the analytical resolution (*p* = 0.527, 0.720, 0.467, and 0.807 for 20, 30, 40, and 50 kb tolerances, respectively; Figures S7 and S8; Table S3). Furthermore, this comparability was robustly maintained across all 19 individual chromosomes (Figure S7). Collectively, these comprehensive quantifications firmly substantiate our claim that the magnitude of intra‐cultivar haplotype structural divergence genuinely approaches the variation observed between distinct cultivars.

These cultivar‐specific boundaries were primarily generated through structural events such as TAD fusions, splits, and the emergence of novel TADs within homologous regions (Figure 2e,f; Figure S6b–d). For instance, a homologous region on chromosome 5 in TS‐hap1 displayed three *de novo* TADs and a TAD fusion event relative to its counterpart in TS‐hap2 (Figure 2e). Specifically, in a homologous region between TS1 (Chr05: 9.80–12.48 Mb) and TS2 (Chr05: 9.96–12.60 Mb), three TAD insertions and one TAD fusion occurred in TS1 relative to TS2 (Figure 2e). Similarly, in a homologous region between PN1 (Chr02: 6.04–7.58 Mb) and TS1 (Chr02: 6.34–7.70 Mb), PN1 underwent three TAD insertions relative to TS1 (Figure 2f).

To elucidate the functional consequences of this architectural variation, we examined the genes located at these dynamic boundaries. Specifically, genes located at cultivar‐specific boundaries exhibited significantly higher expression levels compared to those at conserved boundaries. This transcriptional upregulation represents a robust biological effect size, with a Log2 fold‐change (Log2FC) of 1.01 and a Cliff's Delta of ‐0.434 (95% CI: [−0.398, −0.468]) in TS, and a Log2FC of 0.85 and Cliff's Delta of ‐0.356 (95% CI: [−0.322, −0.389]) in PN (Figure 2g). This suggests that newly formed TAD boundaries establish unique transcriptional regulatory environments. Furthermore, GO enrichment analysis revealed that DEGs at TS‐specific boundaries were predominantly involved in “embryo development”, “seed development”, and “reproductive system development” (Figure 2h). In contrast, genes at PN‐specific boundaries were mainly enriched in processes such as “methylation”, “regulation of chromosome organization”, and “transcription factor binding”. These findings indicate that alterations in TAD architecture are a key contributor to the formation of cultivar‐specific phenotypic traits in grapevine.

To dissect the molecular mechanisms driving these functional differences, we compared the epigenetic features of conserved vs. cultivar‐specific genes (Table S4). Consistent with this active transcriptional state, cultivar‐specific boundaries also exhibited distinct epigenomic features, such as a marked reduction in the proportion of repressive mCHG methylation in both PN (10.9% vs. 14.6%; Δ = −3.7%, 95% CI: [−5.8%, −1.5%], Cohen's h = 0.110, *p* = 0.0019) and TS (15.4% vs. 18.6%; Δ = −3.3%, 95% CI: [−6.0%, −0.5%], Cohen's h = 0.087, *p* = 0.030) (Figure S9). In plants, mCHG is a canonical repressive mark maintained by the RNA‐directed DNA methylation (RdDM) pathway to silence TEs and suppress gene expression. The strong enrichment of mCHG on these conserved genes is consistent with their generally lower expression levels, suggesting that stricter epigenetic surveillance is employed to prevent their aberrant transcription and maintain genome integrity. Furthermore, in the TS cultivar, specific boundaries showed a highly significant enrichment of active gbM marks (51.6% vs. 44.3%; Δ = +7.3%, 95% CI: [3.5%, 11.1%], Cohen's h = 0.147, *p* = 1.28e‐04), whereas gbM levels were comparable in PN (Δ = −1.3%, *p* = 0.438) (Figure S9). Together, these quantitative metrics demonstrate that haplotype‐specific boundaries harbor a more transcriptionally permissive methylation landscape.

### 2.3 Distinct TAD State Transitions Between Haplotypes

**FIGURE 3:**
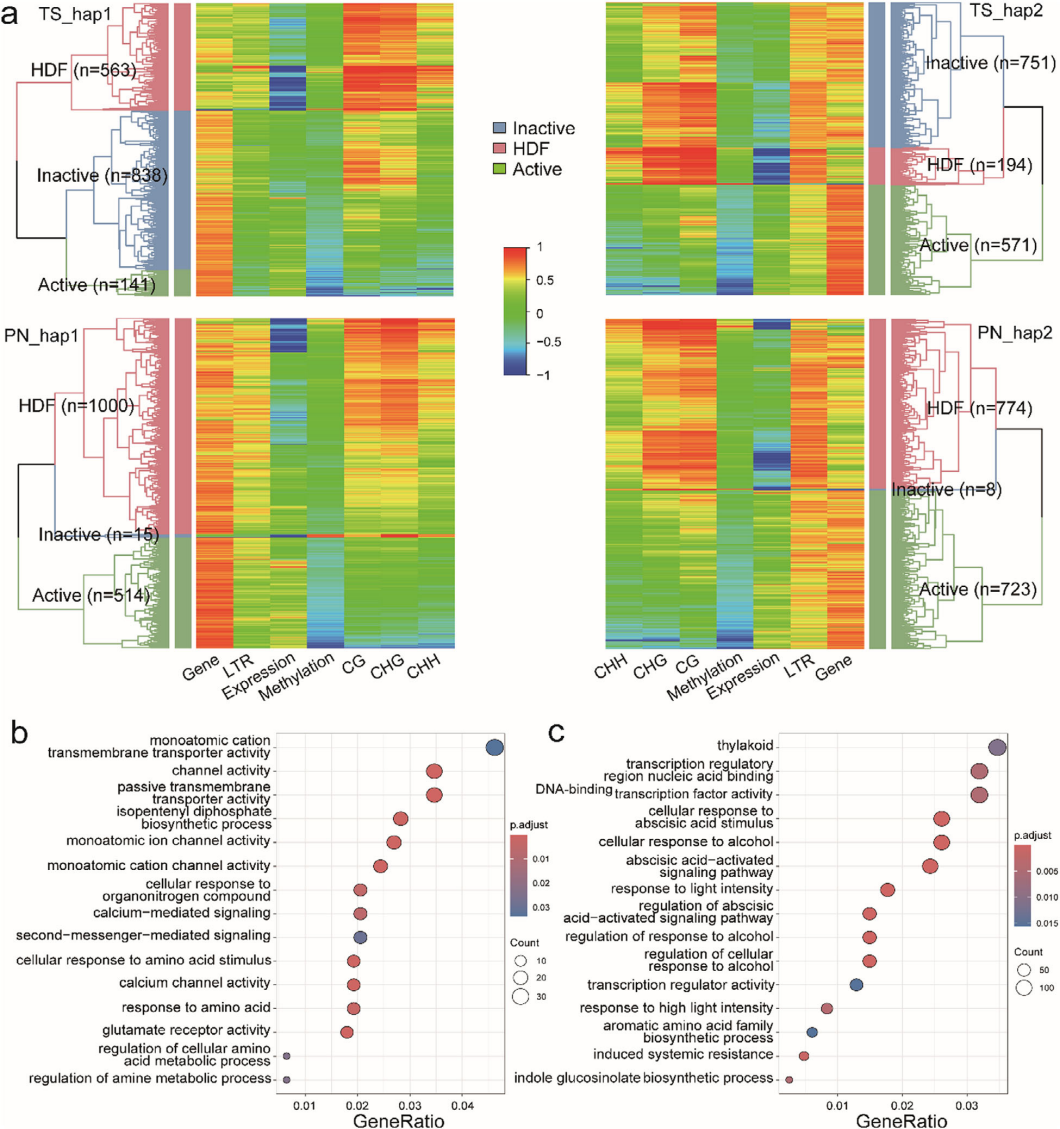
Distinct TAD state transitions between haplotypes. (a) Hierarchical clustering analysis based on genomic and epigenomic features. Three types: active and inactive TADs, heterochromatin‐driven folding (HDF). (b) GO enrichment results of HDF group‐specific genes in TS_hap1. (c) GO enrichment results of active group‐specific genes in TS_hap2.

To understand the nature of TAD dynamics, we classified TADs into three main types based on their genomic and epigenetic features: (1) Active TADs, with high gene density and expression; (2) Inactive TADs, with intermediate features; and (3) Heterochromatin‐Driven Folding (HDF) domains, enriched in LTR retrotransposons and high DNA methylation (Figure 3a; Figures S9 and S10; Table S5; Dataset S1)

Remarkably, we observed large‐scale haplotype‐specific transitions between TAD domain types. In both TS and PN, the hap1 genome was predominantly composed of inactive and HDF domains. These two types constituted 90.9% of all TADs in TS_hap1 (838 inactive, 563 HDF) and 66.4% in PN (15 inactive, 1000 HDF). In contrast, TS_hap2 exhibited a marked shift towards active domains (571), the proportion of active TADs increased by 28.5%, while that of HDF domains (194) correspondingly decreased by 23.7%. Similarly, PN_hap2 (723) increased by 14.4% (from 33.6% to 48.0%) as HDF TADs (774) decreased by 14.0% (Figure 3a). These observations highlight extensive haplotype‐specific chromatin activation.

Genes in the newly activated TADs of TS_hap2 were enriched for transcriptional regulation, including abscisic acid signaling pathways, while the remaining HDF‐specific genes in TS‐hap1 were related to metabolism (Figure 3b,c). As expected, active domains largely overlapped with ‘A’ compartments and HDF domains with ‘B’ compartments, and TAD boundaries were significantly enriched at compartment borders, indicating a strong interplay between these layers of organization (Figures S10, S11a, S12a, and S13). In addition, several large TADs were observed spanning heterochromatic regions but flanked by transcriptionally active domains (Figures S11b, S12b, and S13). Given that HDF domains account for a substantial proportion of TADs in grapevine (ranging from 12.8% to 65.4% across haplotypes), heterochromatin folding may also contribute to TAD organization in grapevine.

### 2.4 Transcriptional Consequences of Haplotype‐Specific TAD State Transitions

To investigate the functional consequences of haplotype‐specific chromatin architecture, we first identified homologous gene pairs and assessed their differential expression between haplotypes in TS and PN. In TS, we detected 14,970 homologous gene pairs were located within TADs, including 114 up‐regulated genes and 85 down‐regulated genes. In PN, we detected 15,477 homologous genes pairs located in TADs, including 118 up‐regulated genes and 95 down‐regulated genes (Table S6).

**FIGURE 4:**
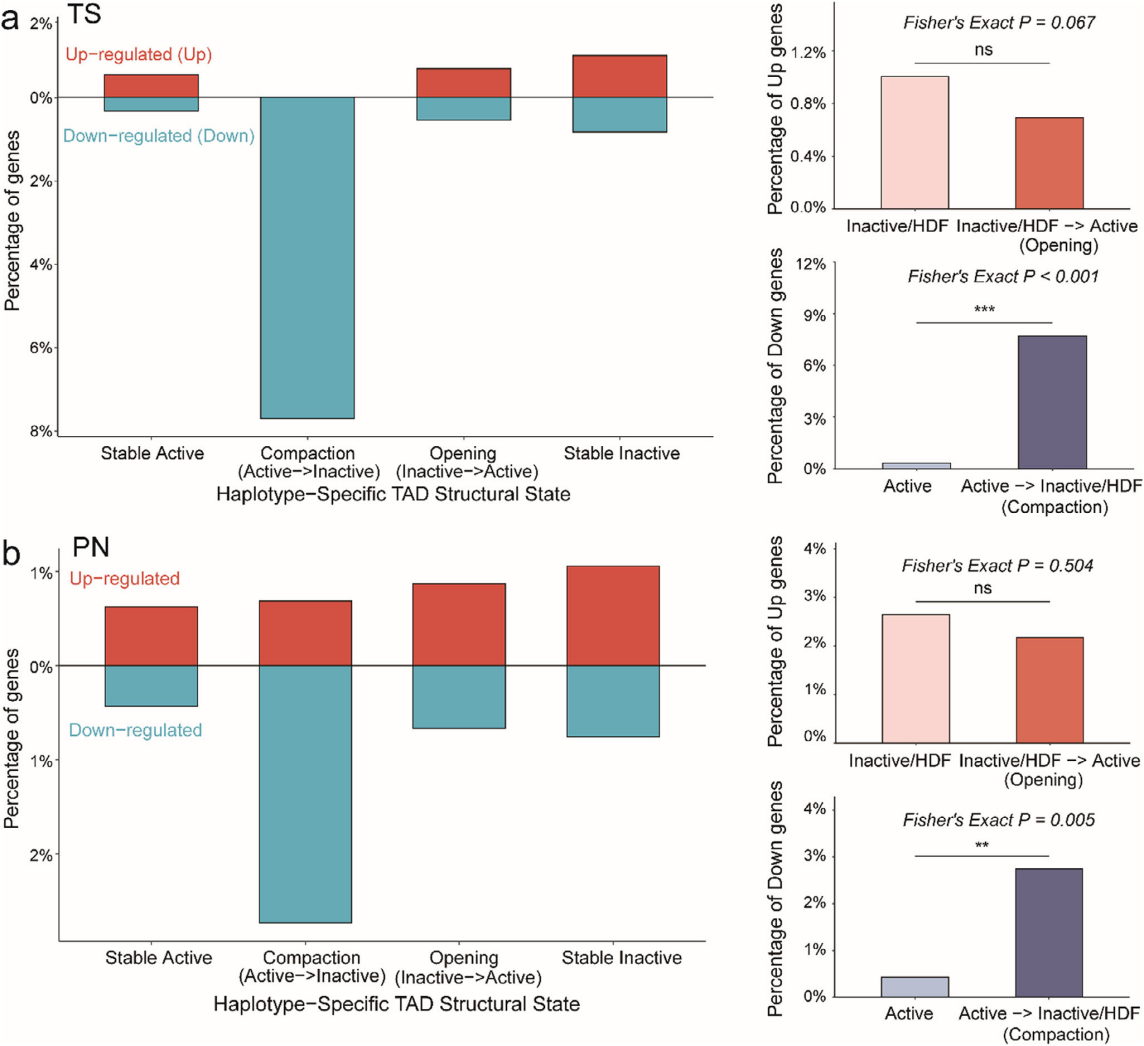
Association between haplotype‐specific TAD structural state transitions and differential gene expression. (a) Distribution of differentially expressed genes (DEGs) across haplotype‐specific TAD structural states in the TS cultivar. TADs were classified into four categories according to their structural states between haplotypes: stable active, compaction (Active→Inactive/HDF), opening (Inactive/HDF→Active), and stable inactive. The bar plots show the percentage of up‐regulated genes (red) and down‐regulated genes (blue) located within each TAD category. Down‐regulated genes are plotted as negative values for visualization. (b) Same analysis as in (a) for the PN cultivar. Right panels show enrichment tests for genes associated with specific TAD structural transitions. The upper panels compare the proportion of up‐regulated genes located in regions undergoing chromatin opening (Inactive/HDF→Active) relative to genes located in inactive/HDF regions, while the lower panels compare the proportion of down‐regulated genes located in regions undergoing chromatin compaction (Active→Inactive/HDF) relative to genes located in active regions. Percentages represent the fraction of genes in each category relative to the total number of analyzed genes. Statistical significance was evaluated using two‐sided Fisher's exact test. Significance levels are indicated as follows: ns, not significant; ^**^, *p* < 0.01; ^***^, *p* < 0.001.

To systematically determine whether these expression divergences were driven by haplotype‐specific TAD state transitions, we decoupled the structural shifts into chromatin compaction (Active→Inactive/HDF) and chromatin opening (Inactive/HDF→Active). Strikingly, our analysis revealed a profound and highly asymmetric transcriptional response to 3D structural reorganization that was remarkably consistent across both cultivars (Figure 4).

Chromatin compaction exerted a massive, dominant repressive effect on local transcription. For genes residing in structurally stable active TADs, the baseline rate of transcriptional down‐regulation was minimal in both TS (0.43%, 39 out of 8,982 genes) and PN (0.33%, 11 out of 3316 genes). In stark contrast, for genes located within TADs undergoing chromatin compaction (Active → Inactive/HDF), the proportion of significantly down‐regulated genes surged to 2.74% in TS (4 out of 146 genes) and 7.69% in PN (3 out of 39 genes). This represents an approximately 6.3‐fold and 23‐fold enrichment of gene silencing triggered by TAD compaction in TS and PN, respectively, both of which are highly statistically significant (Fisher's Exact *p* < 0.001 for TS; *p* = 0.005 for PN) (Figure 4 and Table S7).

Interestingly, the reverse structural transition, chromatin opening, did not yield a symmetric surge in gene activation. The proportion of up‐regulated genes within TADs undergoing structural opening (Inactive/HDF→Active) was extremely low in both TS (0.87%, 22 out of 2534 genes) and PN (0.69%, 48 out of 6952 genes). These rates were statistically indistinguishable from the baseline biological noise observed in structurally stable inactive TADs for TS (1.06%, 35 out of 3308 genes; *p* = 0.067, ns) and PN (1.01%, 52 out of 5170 genes; *p* = 0.504, ns) (Figure 4 and Table S7).

Together, these quantitative results highlight a notable asymmetry in the relationship between 3D genome reorganization and transcriptional output. While chromatin compaction is tightly associated with allele‐biased gene silencing, structural opening alone appears to establish a transcriptionally permissive state rather than directly driving gene activation. This suggests that transitioning to an active topological state may provide the necessary spatial accessibility, but actual transcriptional up‐regulation likely requires additional regulatory layers, such as the recruitment of specific transcription factors. Importantly, these correlative observations perfectly align with the current paradigm of chromatin regulation, where topological opening serves as a permissive foundation for transcription [27], whereas heterochromatin compaction, often shaped by structural variants like TE insertion, acts as a robust silencing mechanism in plant genomes [28, 29].

### 2.5 Structural Variants Shape Haplotype‐Specific 3D Genome Architecture

**FIGURE 5:**
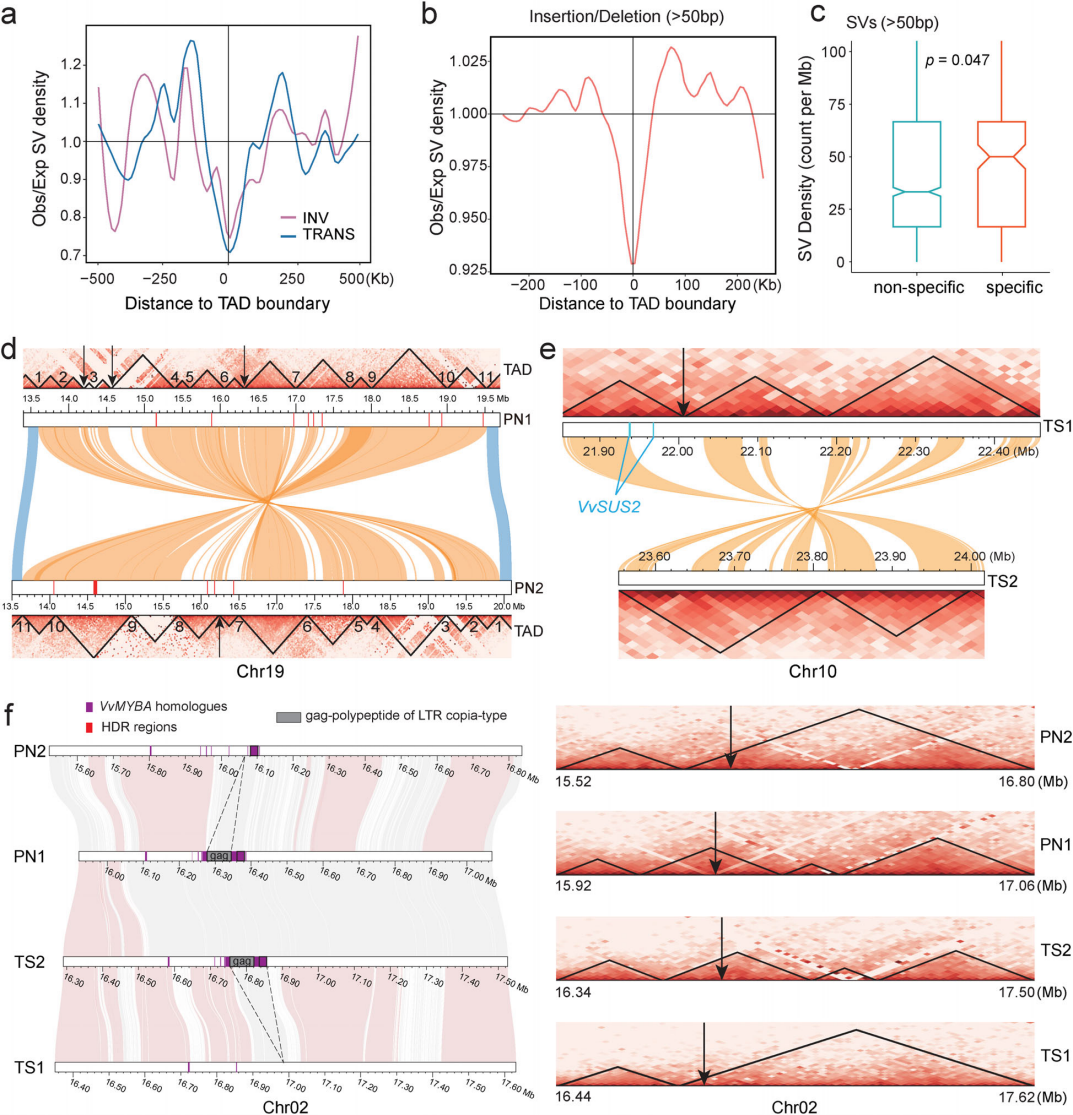
Effects of SVs on 3D genome and key agronomic trait. (a) Comparison of observed (Obs) and expected (Exp) distributions of INV and TRANS near TAD boundaries. (b) Comparison of Obs and Exp distributions of large SVs (> 50 bp) near TAD boundaries. (c) SV densities at the boundaries of conserved and specific TAD boundaries. Statistical significance was determined using the Wilcoxon rank‐sum test. (d) INV region between PN_hap1 and PN_hap2 and its surrounding TAD structure. (e) Co‐linear characterization of the region near the *VvSUS2* gene and its TAD structure in two TS haplotypes. (f) Characterization of the position, structure of the *VvMYBA* gene and its surrounding TAD organization in four haplotype genomes.

To understand the genomic basis for the observed divergence in 3D genome architecture, we investigated the role of SVs. A pile‐up analysis revealed that TAD boundaries are generally regions of high evolutionary constraint, showing a significant depletion of both single nucleotide polymorphisms (SNPs) and large SVs compared to their flanking regions (Figure 5a,b; Figure S14). This underlying stability highlights the functional importance of TAD boundaries in genome organization.

However, a direct comparison between conserved and haplotype‐specific boundaries revealed a starkly different pattern. Haplotype‐specific boundaries were enriched in SVs, indicating that while most boundaries are stable, the turnover and formation of novel boundaries are strongly associated with the occurrence of structural variation (Figure 5c). A clear example of this principle is a large inversion on chromosome 19 in PN, which is directly associated with the formation of multiple new TAD boundaries in PN‐hap1 relative to PN‐hap2 (Figure 5d).

Our results provide a nuanced view of the relationship between genomic variation and TAD organization. At first glance, our finding that TAD boundaries are regions depleted of SNPs and SVs seems to contradict reports of SVs facilitating the emergence of new boundaries [21]. However, we resolve this apparent paradox by proposing a dual‐role model for TAD boundaries in genome evolution. On one hand, the core regions of established, conserved TAD boundaries act as critical insulators and are under strong purifying selection to maintain chromatin integrity, thus repelling most mutations [30]. On the other hand, the turnover of these boundaries, the very process of their formation and disruption, is closely linked to SV dynamics. Supporting this, we found that haplotype‐specific boundaries, hotspots of 3D genome innovation, were significantly more enriched with SVs than conserved boundaries. Therefore, TAD boundaries are simultaneously zones of high constraint and focal points of architectural evolution, with SVs serving as major correlates of structural change [31].

### 2.6 Phased 3D Genome Variations are Linked to Key Agronomic Traits

To investigate the functional impact of these phased 3D genome variations, we analyzed their connection to two critical agronomic traits that differ between the cultivars: seedlessness and berry color. In TS, the seedless phenotype is linked to complex structural variations on chromosome 10 [11]. Our analysis revealed that a haplotype‐specific, *de novo* TAD is formed in TS‐hap1, which is absent in TS‐hap2 (Figure 5e). This new TAD boundary is located near the hemizygous gene *VvSUS2*, which is present only in TS‐hap1 and is associated with seed development [11]. This entire region, which also contains other seed‐related genes like *TT16* and *ABS*, is part of a ∼4 Mb inversion, with chromosomal breakpoints concentrated near the novel TAD boundary (Figure 5e; Figure S15). Based on these integrated findings, we hypothesize that the large‐scale inversion created the new TAD boundary, which in turn altered the local regulatory environment, impacting gene expression and contributing to the seedless trait.

The regulation of berry color, controlled by the *VvMYBA* gene family [1, 32, 33], provides a powerful example of how an SV can simultaneously alter local 3D architecture and gene expression. The haplotypes carry different copy numbers of *VvMYBA* homologs (7 in PN‐hap1, 8 in PN‐hap2, 2 in TS‐hap1, and 5 in TS‐hap2) (Table S8). However, a key regulatory event is a retrotransposon insertion upstream of the *VvMYBA1* gene. This insertion, present in PN‐hap1 and TS‐hap2, is directly associated with the creation of a new, haplotype‐specific TAD boundary and, concurrently, the silencing of the adjacent *VvMYBA1* allele (Figure 5f). Our result confirmed that this transposon suppresses *VvMYBA1* transcription, consistent with previous findings [34]. This leads to distinct outcomes in each cultivar: In PN, the gene is silenced on PN‐hap1 (due to the transposon) but expressed from PN‐hap2, resulting in monoallelic expression that produces colored berries. In TS, the gene is completely lost on TS‐hap1, and the copy present on TS‐hap2 is silenced by the transposon, thereby ensuring the fruit exhibits a complete lack of color.

Together, these examples from two distinct agronomic traits indicate that heterozygous SVs are strongly associated with 3D genome evolution at the haplotype level. Because these SVs represent fundamental DNA‐level variations, the resulting architectural remodeling likely provides a basal, tissue‐independent physical scaffold. This spatial framework reshapes local chromatin domains and closely correlates with the allele‐biased expression of critical trait‐controlling genes when they are developmentally activated in specific tissues.

## 3 Discussion

Despite substantial gene content differences driven by SVs between haplotypic genomes in diploid clonal crops, their influence on transcription regulation and high‐order chromatin structures is largely unknown. In this study, we integrated 3D genomic, genomic, epigenetic, and transcriptomic analysis to generate haplotype‐resolved 3D genome profiling and investigate their functional implications of two grapevine cultivars, PN and TS, which display contrasting seed traits and berry color. We discovered a profound divergence in 3D chromatin organization between the two haplotype genomes within a single plant, driven by widespread shifts in TAD boundaries and dynamic transitions between chromatin states. Crucially, our analysis of these transitions revealed an asymmetric regulatory logic. While the transition from an ‘Inactive/HDF’ to an ‘Active’ state creates a permissive transcriptional environment associated with gene upregulation, it is the reverse transition, from an ‘Active’ to an ‘Inactive/HDF’ state, that acts as a powerful repressive mechanism strongly associated with gene silencing. Our findings align with previous research in both plants and animals that has established correlations between SVs, TAD integrity, and gene expression [20, 21, 24, 35]. However, our work extends significantly beyond these observations by providing a haplotype‐resolved mechanistic framework. By dissecting the distinct regulatory outcomes of different TAD state transitions, we support a mechanistic model linking phased SVs to the dynamic 3D chromatin landscape and, ultimately, to the regulation of vital agricultural traits, offering an unprecedented level of detail on how allelic variation may contribute to phenotypic diversity in clonal crops.

### 3.1 Widespread and Dynamic 3D Chromatin Architecture Varies Between Haplotypes

Most 3D genome studies in plants rely on mixed genome sequences, obscuring haplotype‐specific differences, particularly in highly heterozygous crops like grapevine [1]. Our construction of haplotype‐resolved 3D genomic landscapes for TS and PN overcame this limitation, revealing significant variations in chromatin organization between homologous chromosomes. At the broadest scale, we identified distinct ‘A’ (euchromatin) and ‘B’ (heterochromatin) compartments, a feature consistent with observations in other large‐genome plants [16, 28, 35, 36]. While the overall proportion of these compartments was stable, their arrangement was highly dynamic. We observed compartmental state transitions (‘A’ to ‘B’ or ‘B’ to ‘A’) for approximately 7.0% of homologous genes between the two haplotypes within a single cultivar. Between PN and TS cultivars, 10.1% of homozygous genes underwent A/B compartmental transitions. This divergence was even more pronounced between cultivars, affecting 10.1% of homozygous genes. Critically, these shifts were largely allele‐specific, only a small fraction occurred in concert on both homologous alleles (1.5% from AA to BB and 0.8% from BB to AA), whereas the majority (7.8%) of transitions happened on only one of the two haplotypes. This finding aligns with studies showing large differences in gene content and expression between haplotypes in clonal crops, suggesting the existence of unique, allele‐specific regulatory systems [7].

At a finer resolution, we found that the organization of TADs also contributed significantly to 3D genomic variation. Since the turnover of TAD boundaries can indicate structural reorganization [37], we compared them across the four haplotype genomes. This revealed that the proportion of haplotype‐specific TAD boundaries within a single diploid individual was remarkably high (18.5%~23.0%). Strikingly, this profound intra‐cultivar divergence completely approached and overlapped with the boundary variation observed between the two distinct cultivars (18.7%~21.6%) (Figure 2d, Table S3). In‐depth analysis indicated that these haplotype‐specific boundaries primarily arose from TAD fusion and insertion events (Figure 2e,f), mechanisms previously documented in cotton [37]. Together, these findings of pervasive structural differences at both the compartment and TAD levels emphasize the necessity of haplotype‐resolved analysis. Our discovery that genes transitioned from ‘Inactive/HDF’ to ‘Active’ domains demonstrates the widespread creation of a permissive landscape. This suggests that one haplotype can accumulate changes that poise entire chromosomal domains for activation, creating a reservoir of regulatory potential that can be exploited by subsequent mutations or environmental cues.

### 3.2 From 2D to 3D and its Impacts on Genetics and Breeding of Clonal Crops

Previous research has predominantly relied on 2D genomics to explore the genetic features impacting agronomic traits in clonal crops. This perspective has been invaluable, revealing dramatic differences in gene content and expression between haplotypic genomes [1, 5, 7]. Such haplotype‐specific gene activity has been directly linked to key traits, including sex determination [1, 8, 9], berry color [1, 10], and seed traits [11], often associated with dynamic epigenetic modifications like DNA methylation [7]. Yet, this 2D perspective is incomplete, as gene expression ultimately requires precise regulation within the 3D chromatin structure, involving complex interactions between enhancers, promoters, and transcription factors [12, 13, 14].

In this study, by integrating 2D and 3D genomic analyses, we uncovered how this higher‐order structure contributes to genetic and agronomic diversity. We found that transcriptionally repressed regions, enriched in repetitive sequences and high DNA methylation, spatially alternated with active chromatin regions, a conserved principle of chromatin organization observed in both plants and animals [12, 38]. The critical discovery, however, was the significant divergence in the distribution of these active and inactive TAD types between the haplotypic genomes. This highlights that haplotype‐specific chromatin dynamics play a pivotal role in phenotypic diversity. For instance, in the TS cultivar, a remarkable 28.5% of HDFs on one haplotype were converted to active TADs on the other. Genes within these TS_hap2‐specific active TADs were functionally enriched in transcriptional regulation, including the abscisic acid‐activated signaling pathway (Figure 3c), demonstrating a direct link between 3D structural transitions and specific biological processes.

Our study further reveals that SVs serve as a fundamental genetic basis for this 3D genome reorganization, particularly at TAD boundaries. While TADs in animals are highly conserved over millions of years [15, 39], TAD‐like domains in plants exhibit greater evolutionary plasticity, largely due to the rapid evolution of TE‐rich heterochromatin. However, within a species, these boundaries are functionally constrained regulatory hotspots. This is evidenced by the enrichment of both large SVs and small presence/absence variations (PAVs) at TAD boundaries, a pattern consistent with observations in cotton [30, 40, 41]. Factors such as gene density, epigenetic marks, and TEs are key to TAD formation [15, 23, 42], with reverse transcription TEs being particularly enriched in grapevine TAD‐like domains, implicating them as potent agents of 3D chromatin rearrangement [23, 37, 43].

Ultimately, our work provides novel insights into how this SV‐associated 3D architecture may influence candidate genes for key agronomic traits. For example, genes undergoing A/B compartment transitions between grapevine cultivars were enriched in the gibberellin pathway, a critical regulator of seed development. A notable case is the hemizygous *VvSUS2* gene, associated with seed traits, which is localised within *de novo* formed haplotype‐specific TAD regions coinciding with chromosomal breakpoints. In another powerful example, a transposon insertion upstream of the berry color determinant gene *VvMYBA1* in PN_hap1 corresponds to an alteredthe local TAD structure, creating two additional boundaries not present in other haplotypes. This structural modification was directly associated with the differential expression of *VvMYBA1*, supporting a mechanistic model consistent with earlier findings [34]. Together, these results suggest that SVs are strongly associated with reshaping of the 3D genome and altered the spatial configuration and expression of nearby genes, which may contribute to drive phenotypic diversity. While alternative explanations such as independent trans‐acting factors or distinct epigenetic pathways cannot be fully ruled out at this correlative stage, this study underscores the 3D chromatin architecture is a critical component linked to genetic variation, holding significant promise for advancing precision breeding strategies in clonal crops.

## 4 Experimental Section

### 4.1 Plant Materials and Multi‐Οmics Data Generation

This study utilized two grapevine cultivars, PN (black, seeded berries) and TS (white, seedless berries). The haplotype‐resolved genomes and raw Hi‐C sequencing reads for both cultivars were obtained from previously published studies [4, 7, 11].

To investigate the interplay between 3D genome architecture, epigenetics, and transcription, we generated new multi‐omics data from the young leaves of TS and PN. Leaf samples were collected from Agricultural Genomics Institute at Shenzhen, Chinese Academy of Agricultural Sciences. The plant material was collected from the Agricultural Genomics Institute at Shenzhen, Chinese Academy of Agricultural Sciences. For these samples, we prepared and sequenced two Bisulfite‐seq (BS‐seq) and three RNA‐seq libraries as biological replicates, following previously described protocols [7]. All raw sequencing reads were preprocessed using Trimmomatic (v0.38) [44] to remove adapters and low‐quality sequences.

### 4.2 Hi‐C Data Processing and Normalization

To resolve the 3D genome architecture at the haplotype level for the two grapevine cultivars, PN and TS, high‐throughput chromatin conformation capture (Hi‐C) data were processed. To ensure the robustness and reproducibility of the findings, the data were analyzed in parallel using two independent and established pipelines: HiC‐Pro (v2.11.1) [45] and HiCExplorer (v3.6) [46].

Within the HiC‐Pro pipeline, paired‐end reads were mapped to their respective haplotype‐resolved reference genomes using Bowtie2 (v2.5.1) [47]. Following alignment, reads that were unmapped, singletons, or mapped to multiple locations were discarded. The remaining high‐quality alignments were assigned to MboI restriction fragments, and valid interaction pairs were identified after filtering for experimental artifacts such as dangling ends, self‐circles, and PCR duplicate.

Filtered valid read pairs were binned to generate raw interaction matrices at multiple resolutions (10, 20, 40, 100, and 500 kb). These raw matrices were then normalized using the Iterative Correction and Eigenvector decomposition (ICE) method to correct for known experimental biases, such as fragment length, GC content, and mappability [48]. The quality and effective resolution of the Hi‐C data were confirmed at 10 kb resolution, where over 80% of bins contained at least 1000 contacts, consistent with established standards [49]. For downstream analysis and visualization, the ICE‐normalized matrices were converted into multiple formats. We used the hicConvertFormat command within HiCExplorer to generate. h5 and. cool files, while Juicer tools (v1.22.01) [50] were used to produce. hic files for interactive visualization in Juicebox (https://aidenlab.org/juicebox/). Visual comparison of the contact maps produced through these parallel formats revealed high concordance, confirming the robustness of the data for subsequent analysis of 3D chromatin architecture.

### 4.3 Reproducibility Analysis

To assess the reproducibility of the Hi‐C experiments, we performed a correlation analysis between the two biological replicates. The analysis was conducted using the hicCorrelate tool from the HiCExplorer suite on ICE‐normalized contact matrices at 20 kb resolution. Prior to the calculation, contact frequencies were log1p‐transformed to stabilize variance. The analysis was restricted to intra‐chromosomal contacts with a genomic distance ranging from 5 to 200 kb to focus on meaningful chromatin interactions. The biological replicates exhibited high concordance, with a Pearson correlation coefficient of 0.93 (Figure S16), confirming the high quality and reliability of the data for subsequent 3D genome analysis.

### 4.4 Identification of A/B Compartments and Subcompartments

To ensure a highly reliable demarcation of chromatin compartments, a consensus approach was employed, integrating three independent computational methods. The primary identification of A/B compartments was performed on the ICE‐normalized Hi‐C contact matrices at 100‐kb resolution using the cooltools eigs‐cis function [51]. This tool utilizes eigenvector decomposition to derive the first three eigenvectors (E1, E2, E3). To robustly identify the eigenvector that best represented the A/B compartmentalization (typically E1), we manually inspected the correlation of each eigenvector with chromosome‐wide gene density. The selected eigenvector was then oriented such that positive values, corresponding to gene‐rich regions, were assigned to the active ‘A’ compartment, and negative values were assigned to the repressed ‘B’ compartment.

To resolve finer‐scale chromatin domains, subcompartments were inferred using Calder [52] on the 100‐kb resolution Hi‐C matrix. With default parameters, Calder partitioned the genome into 2, 4, and 8 subcompartments at three hierarchical levels, providing a multiscale view of chromatin organization that correlated strongly with genomic and epigenomic features. As a third, complementary approach, Principal Component Analysis (PCA) was performed using HOMER [53] on the chromatin interaction matrix. The first principal component (PC1) was used to distinguish A/B compartments, with the sign of PC1 values manually adjusted based on gene density to ensure consistency. The high concordance in compartment calls across these three distinct methods provided a robust foundation for all subsequent analyses.

### 4.5 Comparative Analysis and Quantification of Compartment Switching

To investigate haplotype‐specific differences in chromatin organization within and between the PN and TS cultivars, the compartment status of homologous genes was compared across the four haplotype genomes. One‐to‐one orthologous gene sets were identified using OrthoFinder (v2.3.8) [54].

A gene's compartmentalization was defined as conserved if it was located in the same compartment type (i.e., both in ‘A’ or both in ‘B’) between the haplotypes being compared. Conversely, a compartment switch was defined for any orthologous gene found in an ‘A’ compartment in one haplotype and a ‘B’ compartment in the other (or vice versa). This framework allowed for the systematic quantification of large‐scale chromatin state switching and its association with allele‐specific gene expression.

### 4.6 Functional Enrichment Analysis of Switched Genes

To understand the potential functional consequences of observed compartment switching, Gene Ontology (GO) enrichment analysis was performed on the sets of genes that changed compartment status. First, GO terms were assigned to all protein‐coding genes in the phased genomes using the eggNOG‐mapper web server [55]. Subsequently, GO enrichment analysis for specific gene lists (e.g., genes switching from ‘A’ to ‘B’) was conducted using the R package clusterProfiler (v3.10.1) [56] and corroborated with TBtools [57]. GO terms with a False Discovery Rate (FDR) of less than 0.01 were considered significantly enriched.

### 4.7 TAD Identification

To investigate chromatin organization at a sub‐megabase scale, TADs were identified from the ICE‐normalized Hi‐C contact matrices at 20 kb resolution. We employed the hicFindTADs tool from the HiCExplorer (v3.6) [58], which operates by calculating an insulation score across the genome. This score measures the density of Hi‐C interactions in a local window, where local minima in the score profile, representing regions of depleted cross‐boundary interaction, are used to define TAD boundaries. Default parameters were used to delineate the genome‐wide set of TADs for each of the four grapevine haplotypes.

### 4.8 Feature‐Based Clustering and Characterization of TADs

To classify TADs into distinct functional categories (Active, Inactive, HDF) using reproducible quantitative criteria, we used a data‐driven hierarchical clustering pipeline based on seven comprehensive genomic, epigenomic, and transcriptomic features. Specifically, gene density and transposable element (TE) content were calculated as the fraction of the TAD covered by their respective annotations using bedtools coverage. For transcriptomic features, RNA‐seq reads, cleaned reads were processed with Trimmomatic (v0.38) and aligned with HISAT2 (v2.2.1) [44, 59], after which the transcriptional activity for each TAD was quantified by calculating the average Counts Per Million (CPM) using deepTools (v3.3.0) [60], averaged across three biological replicates. For epigenomic features, DNA methylation levels were assessed using BS‐seq data aligned with Bismark (v0.22.3) and Bowtie2 (v2.5.1) [61]. We calculated the global methylation level, as well as the specific densities of CG, CHG, and CHH methylation (defined as the percentage of cytosine sites within the TAD showing ≥ 25% methylation). Notably, the CG methylation profiles from BS‐seq were highly consistent with independent 5mC profiles called from ONT ultra‐long reads using the Dorado basecaller (model dna_r9.4.1_e8_sup@v3.3) (Pearson's *r* > 0.93, *p* < 0.001; Figure S17).

To account for the varying scales of these diverse features, the raw values were mean‐normalized, log2‐transformed with a pseudocount of 0.01, and min‐max scaled to a uniform range of [−1, 1]. Agglomerative hierarchical clustering was subsequently performed on this normalized feature matrix using the Euclidean distance metric and the complete linkage method (hclust function in R v4.3.1). The resulting dendrogram was strictly cut into exactly three clusters (k = 3), which were biologically annotated as Active, Inactive, and HDF based on their distinct and aggregated multi‐omics profiles.

### 4.9 Comparative Analysis of TAD Architecture

To identify conserved and haplotype‐specific TAD boundaries, we used the Tcbf tool [62]. A boundary was classified as “conserved” between two genomes if it was either flanked by homologous gene blocks (identified via MCScan) [63] or shared significant sequence similarity in the boundary region (identified via minimap2) [64]. To account for minor technical variability in boundary calling, a baseline shift of up to 20 kb (one bin) was initially permitted. Furthermore, to rigorously ensure that our comparative results were not artifacts of this specific analytical window, we performed a comprehensive sensitivity analysis by systematically varying the boundary‐matching tolerance across multiple resolutions (20, 30, 40, and 50 kb). This was executed by adjusting the flanking extraction distance (‘‐d 20 000, 30 000, 40 000, 50 000’) within the ‘tcbf first‐extract‐boundary’ module. This rigorous analysis allowed for the robust identification of structural events such as TAD fusion, splitting, and the emergence of novel TADs, which collectively underpin the dynamic TAD landscape.

### 4.10 Characterization of Genes at Conserved and Specific Loci

To understand the functional impact of TAD boundary dynamics and cultivar‐specific evolution, we characterized the genes located at these loci.

(1) Gene‐body methylation (gbM) classification: We classified genes into four methylation categories (gbM, CHG‐methylated, CHH‐methylated, or unmethylated) based on context‐specific methylation patterns within the protein‐coding region (start to stop codon). Following established methods [7], we used a one‐tailed binomial test to determine if the methylation level of a gene was significantly higher than the genomic average. A gene with at least 20 CG sites was classified as gbM if only its CG context was significantly methylated (*p* ≤ 0.05). Similarly, a gene was CHG‐methylated if its CHG context was significant (*p* ≤ 0.05) but its CHH was not, and CHH‐methylated if its CHH context was significant (*p* ≤ 0.05). Genes lacking significant methylation in all three contexts were classified as unmethylated. Genes with ambiguous patterns were excluded from this analysis.
(2) Gene expression quantification: Gene‐level expression was quantified from RNA‐seq data. High‐quality reads were mapped using HISAT2 (v2.2.1) [59], and raw counts were generated with featureCounts (v2.0.1) [65]. Expression levels were normalized to Fragments Per Kilobase of transcript per Million mapped reads (FPKM). A gene was considered expressed if its FPKM was > 1.

To infer the biological processes associated with these dynamic TAD boundaries, we performed a GO enrichment analysis using the same methodology described in Section 4.6.

### 4.11 Analysis of TAD State Transitions and Gene Expression

To investigate the functional link between chromatin architecture dynamics and transcriptional regulation, we performed a comparative analysis of TAD state transitions and their association with differential gene expression between haplotypes.

For within‐cultivar comparisons, homologous gene pairs between the two phased haplotypes (hap1 and hap2) of PN and TS were identified using a stringent reciprocal best‐hit approach. An all vs. all protein sequence comparison was conducted for each cultivar's complete phased proteome using BLASTP (v2.12.0) [66]. To define a high‐confidence set of homologs, the results were filtered using custom R scripts to retain only reciprocal best hits that met the following criteria: an *E*‐value < 1e^−10^, a sequence identity > 90%, and an alignment quality score > 0.9, where the score was calculated as (alignment length ‐ mismatches) / alignment length.

Each gene within a homologous pair was assigned the state (Active,‘Inactive, or HDF) of its encompassing TAD, allowing us to classify each pair by its TAD state transition. To perform a robust statistical evaluation of chromatin reorganization, HDF domains were merged with general inactive domains to form a unified HDF/Inactive TAD category. This merging strategy is conceptually grounded in the classic A/B compartment binary model of plant 3D genomes, where the inactive (B) compartment biologically encompasses both TE‐rich constitutive heterochromatin (HDF) and repressed euchromatin (Inactive) [28, 67] (Figures S11b and S12b).

Using this standardized binary model, we classified the TAD structural shifts into four groups: Stable Active, Compaction (Active→Inactive/HDF), Opening (Inactive/HDF→Active), and Stable Inactive. The association between structural transitions and gene expression was statistically evaluated in two specific dimensions using 2 × 2 contingency tables:

(1) Compaction vs. Down‐regulation: We compared the proportion of significantly down‐regulated genes within compacted TADs against the baseline down‐regulation rate in structurally stable active TADs.
(2) Opening vs. Up‐regulation: We compared the proportion of up‐regulated genes in opened TADs against the baseline biological noise in structurally stable inactive TADs.
DEGs between haplotypes were identified using DESeq2 (v1.36.0) [68] with criteria of an adjusted padi < 0.05, |log2FC| ≥ 1. The statistical significance of these enrichments was determined using Fisher's exact test, with a *p*‐value < 0.05 considered significant.

### 4.12 Identification of SVs and Enrichment Analysis

To create a comprehensive SV catalog, whole‐genome alignments were performed between all pairs of haplotypes using minimap2 (‐ax asm5) [64] and MUMmer (−c 100 −b 500 −l 50) [69]. The resulting alignments were processed with SyRI (v1.5.4) [70] to identify SNPs, InDels (>50 bp), inversions (INVs), and translocations (TRANSs).

To assess the enrichment of SVs at TAD boundaries, we performed a pile‐up analysis. For each TAD boundary, we defined flanking regions and divided them into bins. We then calculated the density of each SV type within each bin. This observed density was normalized by the genome‐wide average density for that SV type to generate an observed/expected ratio. To account for the different scales of variants, distinct parameters were used: for SNPs and small InDels, we analyzed ±100 kb regions with 1‐kb bins; for large InDels (>50 bp), ± 250 kb regions with 10‐kb bins; and for large‐scale INVs and TRANSs, ± 500 kb regions with 40‐kb bins. The average ratios across all TAD boundaries were then plotted.

### 4.13 Analysis of 3D Genome Variation on Agronomic Traits

To specifically link 3D genome variation to berry traits, we analyzed a publicly available RNA‐seq dataset profiling berry development in PN and TS (NCBI: PRJNA391820). Raw reads were processed with Trimmomatic [44] and aligned to the PNT2T reference genome [71] using HISAT2. Gene expression was quantified as FPKM using FeatureCounts (v2.0.1) [65]. Differentially expressed genes (DEGs) between cultivars at each time point were identified using DESeq2 (v1.36.0) [68] with criteria of an adjusted *padi* < 0.05, |log2FC| ≥ 1.

Genes located within regions of 3D structural variation (e.g., compartment switches or TAD boundary shifts) were identified using BEDTools [72]. GO enrichment analysis for these genes was performed using the R package clusterProfiler (v3.10.1) [56], with significant terms defined by an *FDR* < 0.01.

### 4.14 Statistical Analysis

Details on statistical analyses used in our study, including the statistical tests used and the number of replicates, were provided in the corresponding figure legends. Statistical analysis and data visualization were performed using R (v.4.3.2).

## Author Contributions

Y.Z., Y.P. and X.W. designed the research. Y.P., X.F., and L.Z. wrote the manuscript. X.F., Y.P. and L.Z. performed the analysis. R.Z., Q.X., Y.L., M.D., T.H., G.H., Y.Z., S.Y., R.Y., Z.J., Y.D., Y.X., H.X., X.X., and Y.L. revised the paper. X.F. and L.Z. collected the data.

## Funding

This research was financially supported by the National Natural Science Foundation of China (No. 32372662), the Science Fund Program for Excellent Young Scholars of the National Natural Science Foundation of China (Overseas) to Yongfeng Zhou, the National Key Research and Development Program of China (2023YFD2200700), the Ningxia Key Research and Development Program (2025BBF02019), and the Project of National Key Laboratory for Tropical Crop Breeding (NKLTCB‐RC202501).

## Conflicts of Interest

The authors declare no conflicts of interest.

## Supporting information




**Supporting File 1**: advs75283‐sup‐0001‐SuppMat.docx.

**Supporting File 2**: advs75283‐sup‐0002‐SuppMat‐DatasetS1.xlsx.

**Supporting File 3**: advs75283‐sup‐0003‐SuppMat‐DatasetS2.xlsx.

**Supporting File 4**: advs75283‐sup‐0004‐SuppMat‐TableS1‐S8.xlsx.

## Data Availability

BS‐seq and RNA‐seq data from TS and PN leaf samples were generated and deposited in the NCBI database (PRJNA1221658) and NGDC database (PRJCA061964). Haplotype‐resolved genome assemblies were used for PN (PRJNA1048106) and TS (PRJNA1021353), along with Hi‐C data for PN (PRJNA1048106, PRJNA1178252) and TS (PRJNA1021353), along with RNA‐seq data collected 20 to 50 days after flowering (DAF) over two years for PN and TS (PRJNA391820), were downloaded from the NCBI database.
