# Supplementary material for: Haplotype‐Resolved 3D Genomic Landscapes and Their Impacts on Agronomic Traits in Grapevine
Source: Adv Sci (Weinh). 2026 Apr 15;13(39):e21838. doi: 10.1002/advs.202521838 (PMC13334877; doi:10.1002/advs.202521838)
Supplement: Supplementary file 1 — Supporting File 1: advs75283‐sup‐0001‐SuppMat.docx. [file ADVS-13-e21838-s001.docx]

Supporting Information

**This Supporting Information file includes:**

- Supplementary Figure S1 to S17 (This file)
- Supplementary Table S1 to S8 (Separate EXCEL file)

Table of Contents

Legends for Supplementary Figures S1-S17

Figure S1 Quality control metrics for Hi-C sequencing libraries

Figure S2 Comparison of genome-wide Hi-C maps across phased genomes of grapevine

Figure S3 Comparison of A/B chromatin compartments across phased grapevine genomes

Figure S4 Genomic and epigenomic features of A/B compartments and subcompartments

Figure S5 Functional analysis of genes within dynamic A/B compartment regions

Figure S6 Comparison of TAD compartments in phased genomes

Figure S7 Chromosome-level distribution of haplotype-specific TAD boundary variations

Figure S8 Parameter sensitivity of haplotype-specific TAD boundary variations

Figure S9 Epigenomic feature profiles on conserved and cultivar-specific TAD boundaries

Figure S10 Aggregate profiles of genomic and epigenetic features at structural domain boundaries

Figure S11 Classification and epigenetic characterization of TAD-like domains in the TS cultivar

Figure S12 Classification and epigenetic characterization of TAD-like domains in the PN cultivar

Figure S13 Epigenetic and TE dynamics around chromatin structure

Figure S14 Genomic distribution of SNPs relative to TAD boundaries

Figure S15 Chromosomal synteny and large-scale inversions among four haplotype genomes

Figure S16 Reproducibility of Hi-C biological replicates

Figure S17 Genome-wide concordance of CG methylation levels between BS-seq and ONT sequencing platforms (R9.4.1 flow cells)

Supplementary Tables S1-S8 (detailed in separate EXCEL file)

Table S1 Hi-C library statistics with data processed using HiCExplorer

Table S2 Hi-C library statistics with data processed using HiC-Pro

Table S3 Sensitivity analysis of haplotype-specific TAD boundary variation at varying matching resolutions

Table S4 Distribution of gene body methylated (gbM), unmethylated (UM), and CHG-methylated (mCHG) genes at haplotype-specific and conserved TAD boundaries in the TS and PN cultivars

Table S5 Median values of seven genomic and epigenomic features, including gene and LTR densities, as well as global, CG, CHG, and CHH methylation levels, across Active, Inactive, and HDF TADs

Table S6 Summary of homologous gene pairs and differentially expressed genes (DEGs) in TS and PN cultivars

Table S7 Association between haplotype-specific TAD state transitions and differential gene expression in TS and PN cultivars

Table S8 *MYBA* homolog genes in the phased genomes

Supplementary Dataset S1

A/B compartments in the four haplotype genomes identified by the three software (Calder, Cooltools, Homer)

Supplementary Dataset S2

Hierarchical clustering analysis of TAD-like domains based on genomic and epigenomic features

**
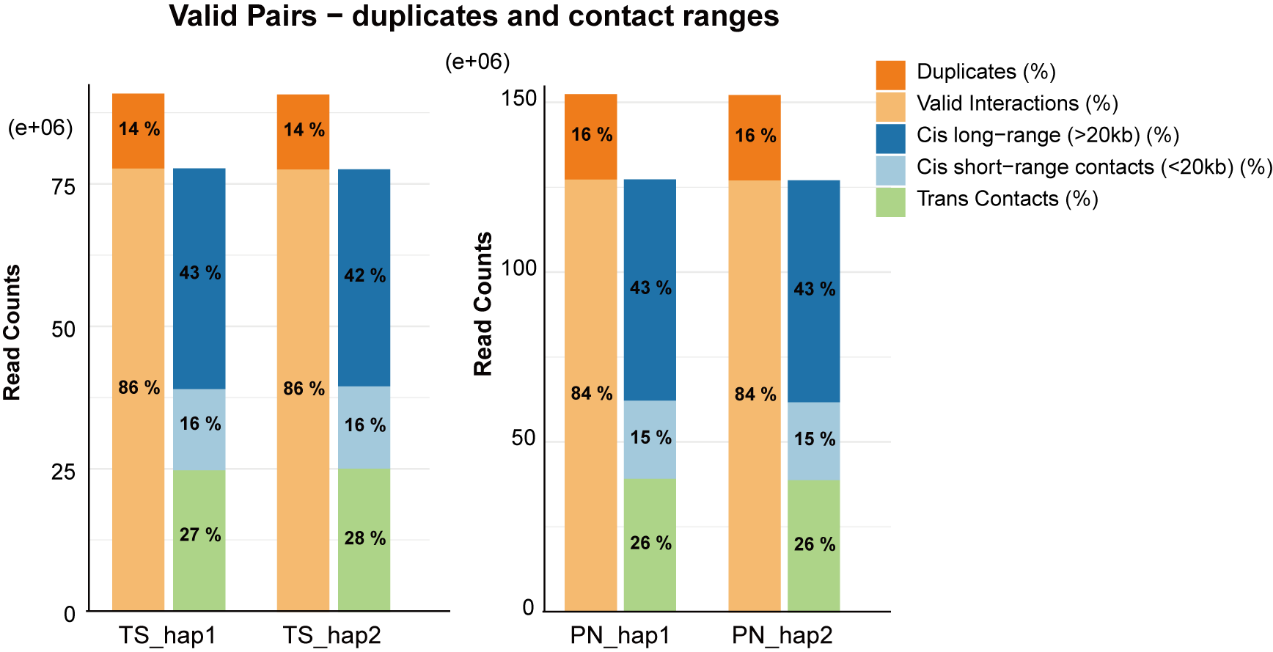
**

**Figure S1. Quality control metrics for Hi-C sequencing libraries.** Quality control (QC) summary for each Hi-C library generated by the HiC-Pro pipeline. The figure displays key mapping statistics, including the fraction of valid interacting pairs and PCR duplicates. It also shows the proportion of intra-chromosomal (cis) versus inter-chromosomal (trans) contacts. For a more detailed view of chromatin proximity, cis contacts are further classified based on their interaction distance into short-range (≤ 20 kb) and long-range (> 20 kb) categories.

**
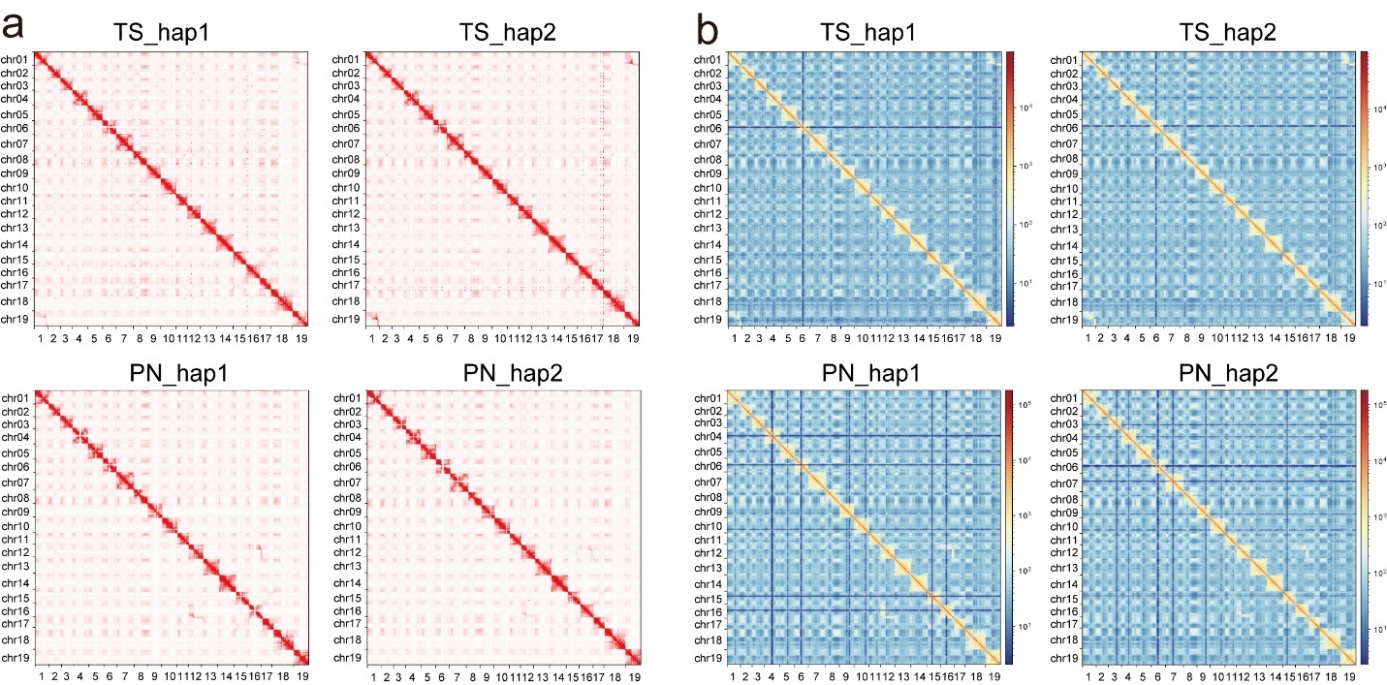
**

**Figure S2. Comparison of genome-wide Hi-C maps across phased genomes of grapevine.** (a) Raw contact matrices at 1 Mb resolution show the initial output from the HiC-Pro pipeline. (b) ICE-normalized and corrected contact maps at 500 kb resolution were generated using HiCExplorer for downstream analysis. The high degree of similarity between the two haplotypes in both raw and normalized maps validates the consistency of the data and the underlying genome phasing.

**
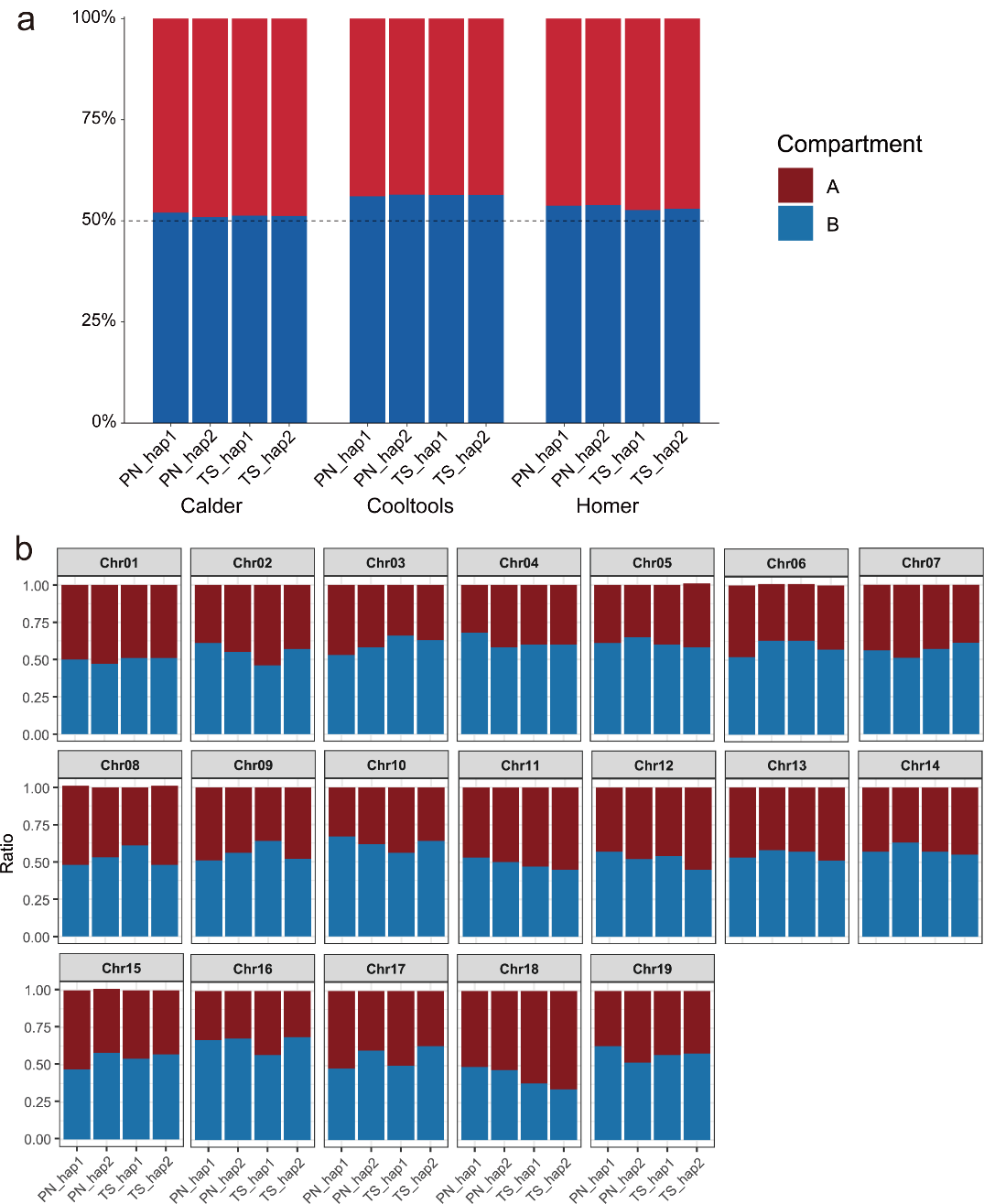
**

**Figure S3. Comparison of A/B chromatin compartments across phased grapevine genomes.** (a) Genome-wide proportions of ‘A’ and ‘B’ compartments identified using three independent Hi-C analysis tools (Calder, Cooltools, and Homer) in four haplotype-resolved grapevine genomes (PN_hap1, PN_hap2, TS_hap1, and TS_hap2). Compartments were defined based on the sign of the first principal component (PC1) derived from eigenvector decomposition of Hi-C contact matrices. Proportions were calculated based on the number of genomic bins assigned to each compartment. The dashed line indicates equal partitioning (50%) between ‘A’ and ‘B’ compartments. (b) Chromosome-level distribution of ‘A’ and ‘B’ compartments across the 19 chromosomes in each haplotype genome. The proportion of bins assigned to ‘A’ or ‘B’ compartments was calculated separately for each chromosome.

**
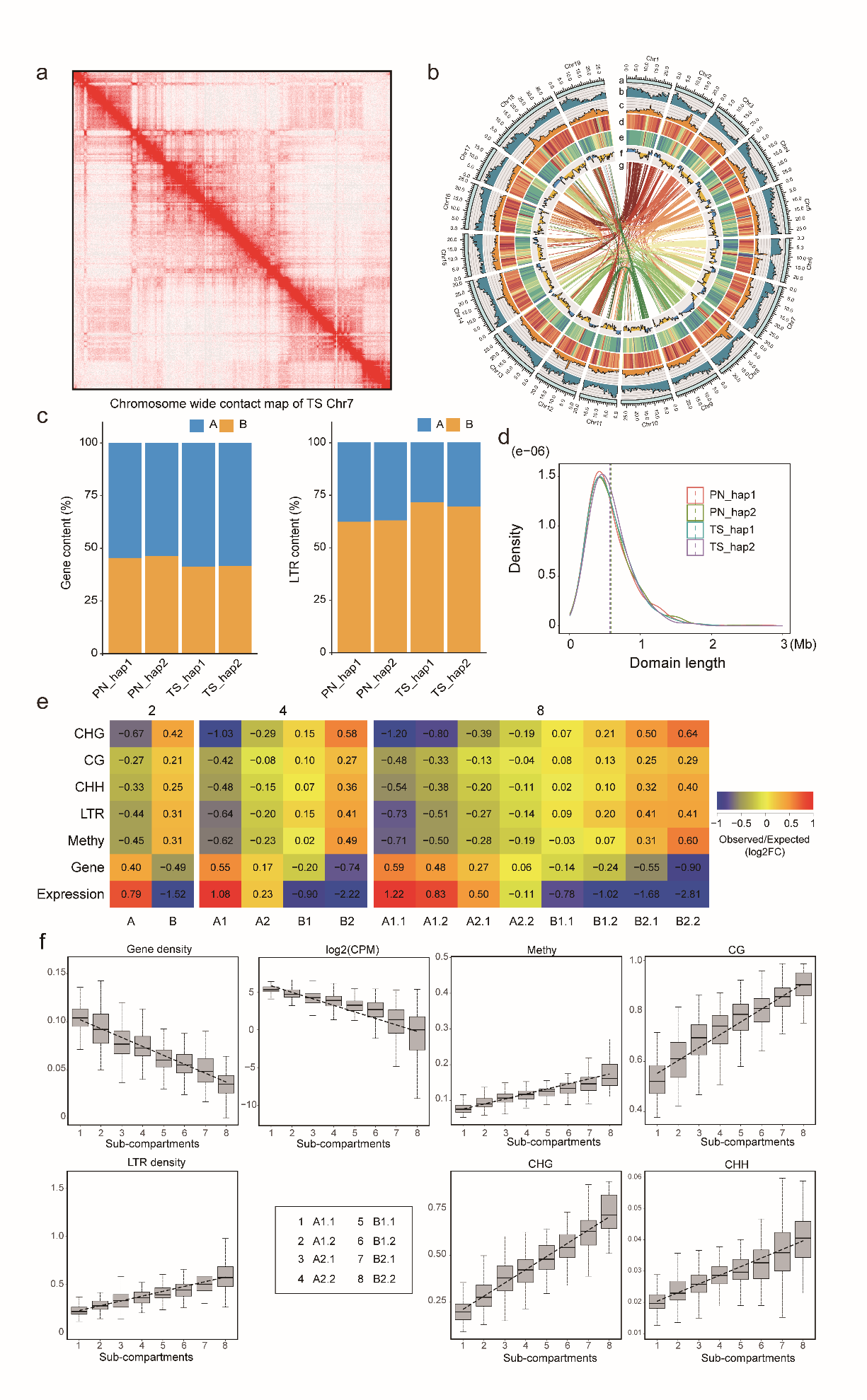
**

**Figure S4. Genomic and epigenomic features of A/B compartments and subcompartments.** (a) Chromosome-wide contact map of Chr7 of TS. ‘A’ compartment is mainly concentrated in the chromosome arm region while ‘B’ compartment is mainly concentrated in the centromere region of chromosome. (b) Circos plot showing chromosome-level features of the TS_hap1. Tracks represent chromosome length (a), Genes (b), LTR retrotransposons (c), GC content (d), gene expression (e), A/B compartment (f), and syntenic blocks (g) in 500 Kb windows. (c) Relative gene content of the A/B compartments in the phased genomes (left). Relative LTR content of the A/B compartments (right). (d) Subcompartment size distribution inferred by Calder using a 100 kb resolution matrix. (e) Characterization of subcompartments in the TS_hap2 genome. (f) Subcompartments are correlated with a number of genomic and epigenomic features in the grapevine genome. Calder-inferred subcompartment ranks are negatively correlated with gene density (left up) and transcription levels (middle) but positively correlated with LTR-RTs density (left low) and DNA methylation level (right). “Combined all sites” indicates all CG and non-CG (i.e. CHG and CHH) sites. The box plots in (f) span from the 25th to 75th percentile, the center lines show the median, and whiskers show maximum and minimum values. Dashed lines represent the fitted linear regression curves.

**
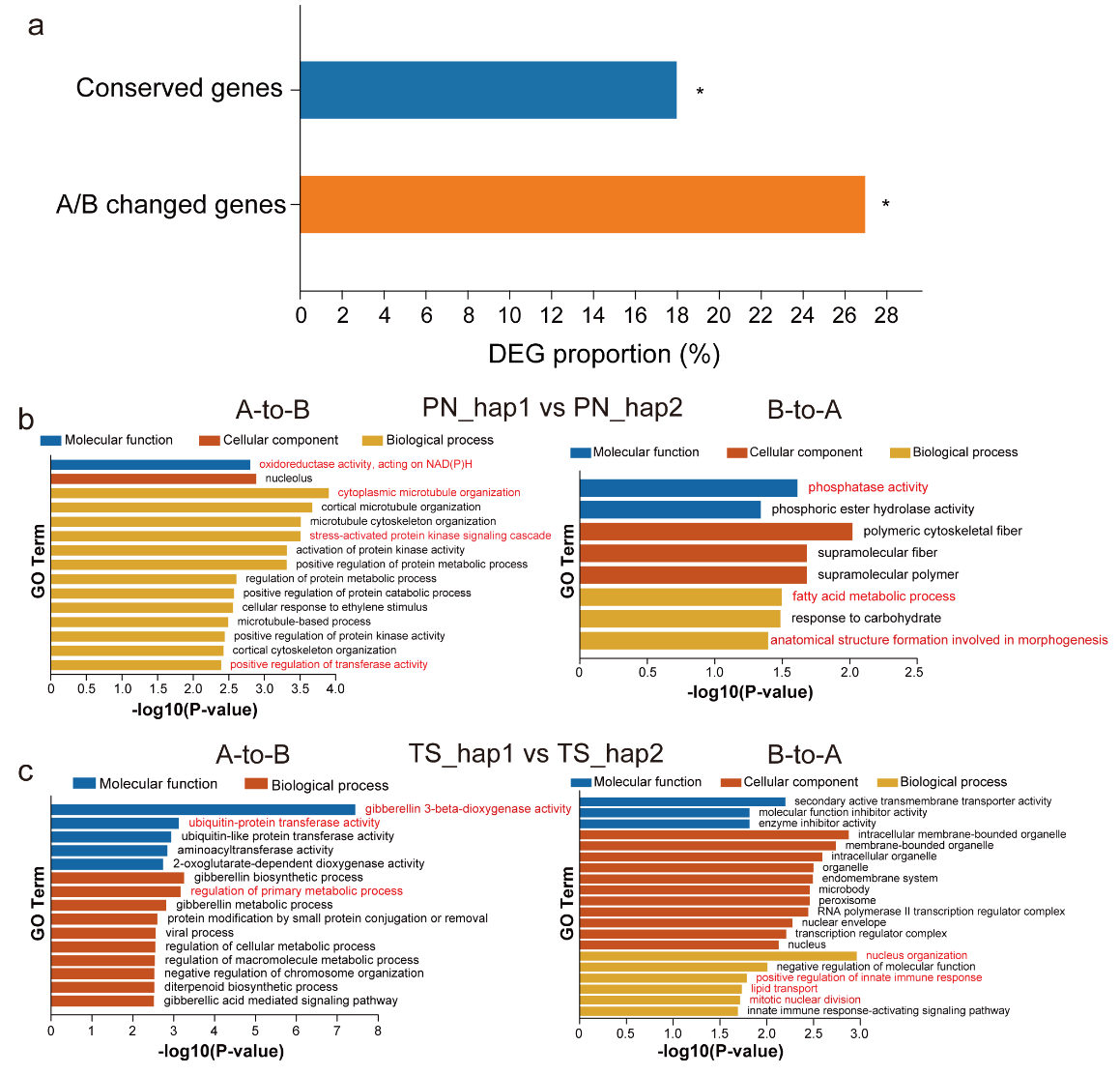
**

**Figure S5. Functional analysis of genes within dynamic A/B compartment regions.** (a) Proportion of differentially expressed genes (DEGs) located in genomic regions with switched A/B compartments versus those in regions with conserved compartments. A/B compartment switching was defined based on changes in the sign of the first principal component (PC1) derived from Hi-C eigenvector decomposition. DEGs were identified using DESeq2 (adjusted *P*-value < 0.05 and |log2FC| ≥ 1). Statistical significance was assessed using a chi-square test. * indicate significant differences (*P* < 0.05). (b, c) Gene Ontology (GO) enrichment analysis for genes located in regions that switched from A-to-B (left panels) or B-to-A (right panels). Enrichment analysis was performed separately for haplotype comparisons within the PN cultivar (b) and the TS cultivar (c). The x-axis represents −log10 (adjusted *P*-value). GO categories are grouped into molecular function, biological process, and cellular component. Only significantly enriched terms (FDR < 0.05) are shown.

**
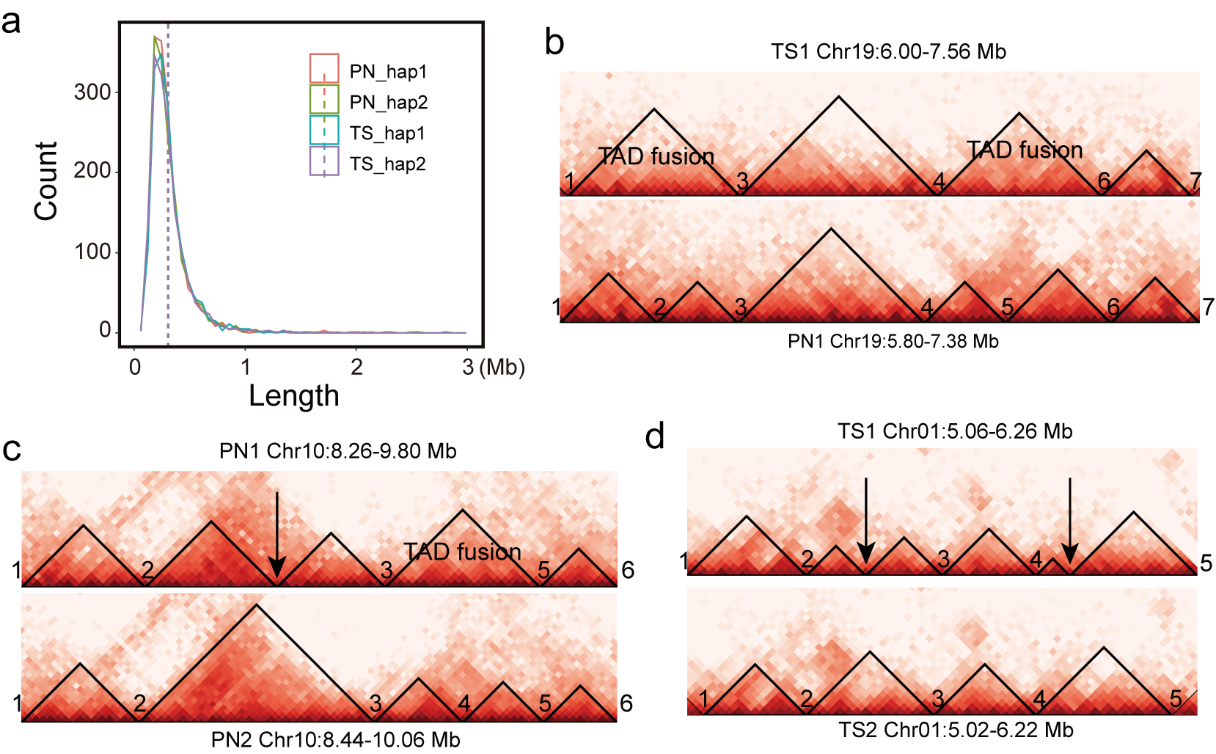
**

**Figure S6. Comparison of TAD organization in phased genomes.** (a) TAD size distribution across the four phased haplotypes. Vertical dashed lines represent the mean TAD size for each respective haplotype. (b) A representative example of TAD structure comparison within a syntenic region of chromosome 19 between two different cultivars (TS_hap1 vs. PN_hap1). (c, d) Representative examples of TAD structure comparison within syntenic regions between the two phased haplotypes of the same cultivar: PN on chromosome 10 (c) and TS on chromosome 1 (d). n the Hi-C heatmaps (b-d), TADs are outlined by black triangles. Conserved TAD boundaries.

**
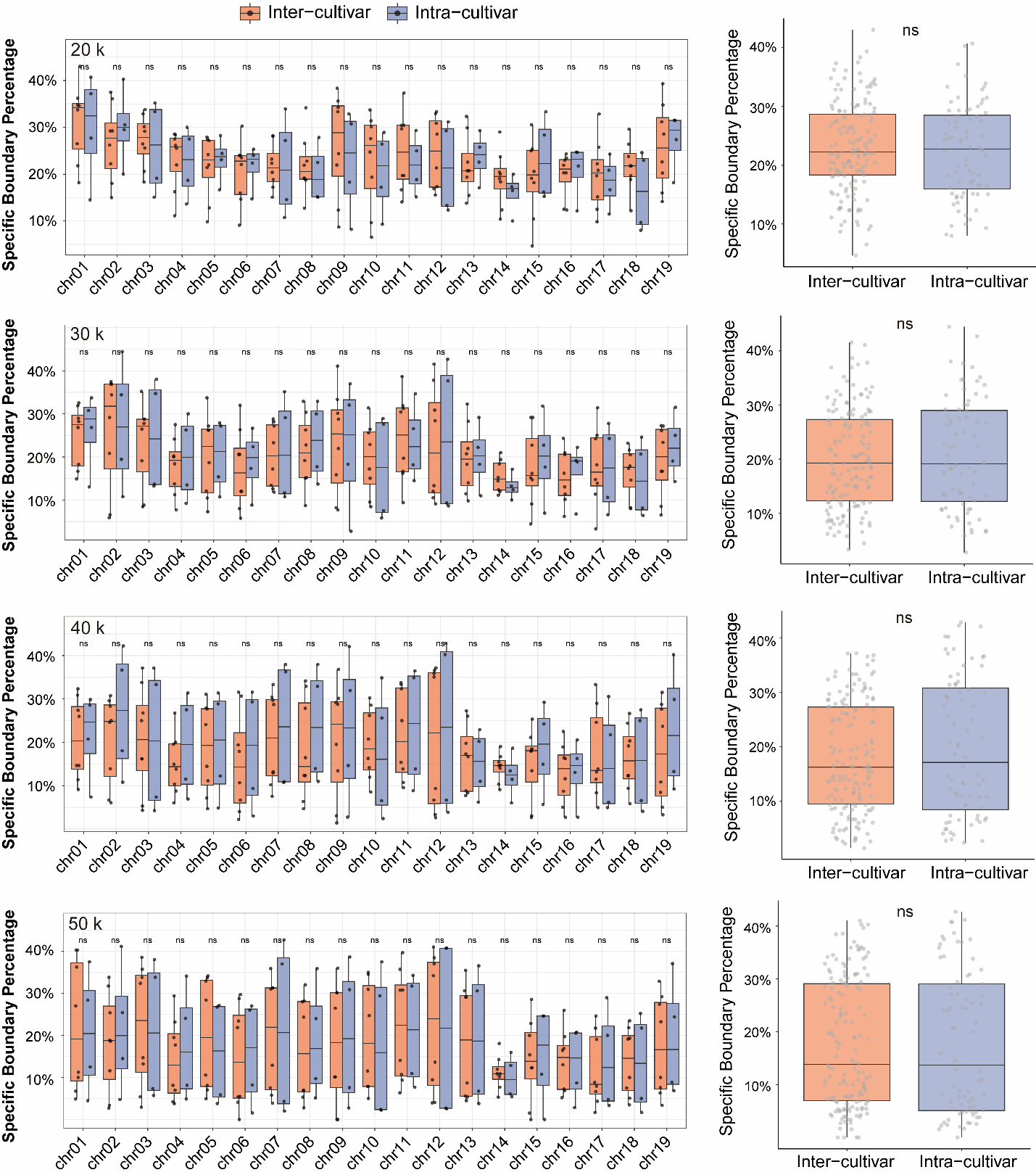
**

**Figure S7. Chromosome-level distribution of haplotype-specific TAD boundary variations.** Grouped boxplots illustrate the percentages of specific TAD boundaries across all 19 chromosomes. Pairwise boundary comparisons are categorized into inter-cultivar (orange) and intra-cultivar (blue) groups. Black dots represent the specific boundary variation rates from individual pairwise comparisons. The data presented in this panel are based on varying boundary-matching tolerances of 20 kb, 30 kb, 40 kb, and 50 kb. Statistical significance between inter- and intra-cultivar rates for each chromosome was evaluated using the Wilcoxon rank-sum test (ns: not significant, *P* > 0.05), confirming that the comparable magnitude of boundary variation between the two groups is a consistent, genome-wide phenomenon.

**
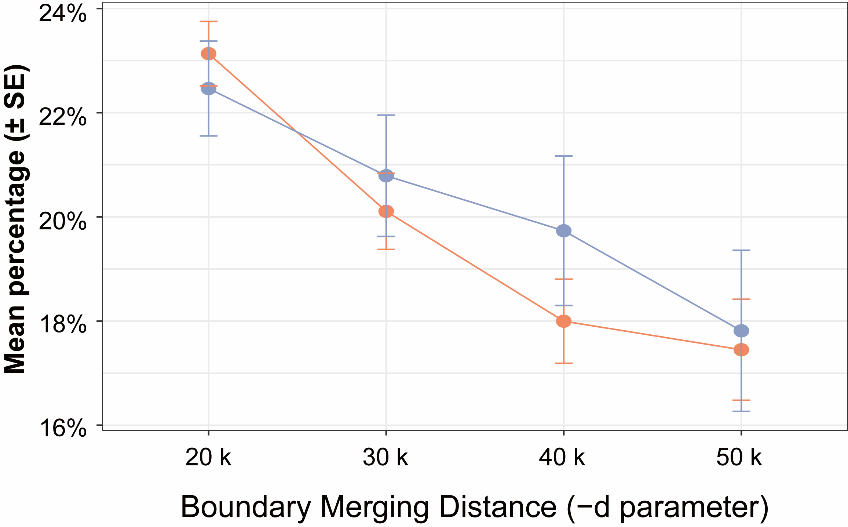
**

**Figure S8. Parameter sensitivity of haplotype-specific TAD boundary variations.** Sensitivity analysis of specific boundary variation rates across varying distance tolerances for boundary matching (-d = 20 kb, 30 kb, 40 kb, and 50 kb in tcbf). The parallel trends demonstrate that the narrow gap between intra- and inter-cultivar variations is biologically robust and not an artifact of a specific parameter choice.

**
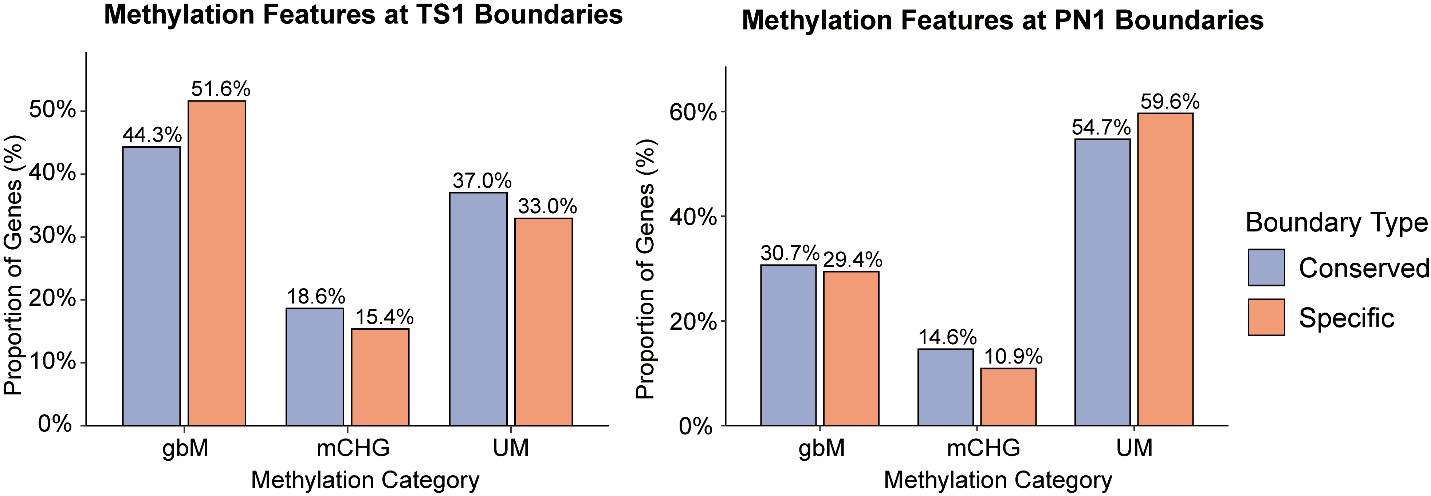
**

**Figure S9. Epigenomic features on conserved and cultivar-specific TAD boundaries.** Proportional distribution of three distinct gene methylation categories, gene body methylation (gbM), CHG methylation (mCHG), and unmethylated genes (UM), located at cultivar-specific versus conserved TAD boundaries. The y-axis represents the percentage of genes within each category. To rigorously quantify the biological magnitude of these epigenomic differences, absolute proportional shifts (Δ%), Cohen’s h effect sizes, and 95% confidence intervals (CIs) were calculated using two-sample test for equality of proportions. Cultivar-specific boundaries consistently exhibited a significantly reduced proportion of repressive mCHG marks compared to conserved boundaries in both PN (10.9% vs. 14.6%; Δ = -3.7%, 95% CI: [-5.8%, -1.5%], Cohen’s h = 0.110, *P* = 0.0019) and TS (15.4% vs. 18.6%; Δ = -3.3%, 95% CI: [-6.0%, -0.5%], Cohen’s h = 0.087, *P* = 0.030). Furthermore, in the TS cultivar, specific boundaries showed a highly significant enrichment of active gbM marks (51.6% vs. 44.3%; Δ = +7.3%, 95% CI: [3.5%, 11.1%], Cohen’s h = 0.147, *P* = 1.28e-04), whereas gbM levels were comparable in PN (Δ = -1.3%, *P* = 0.438).

**
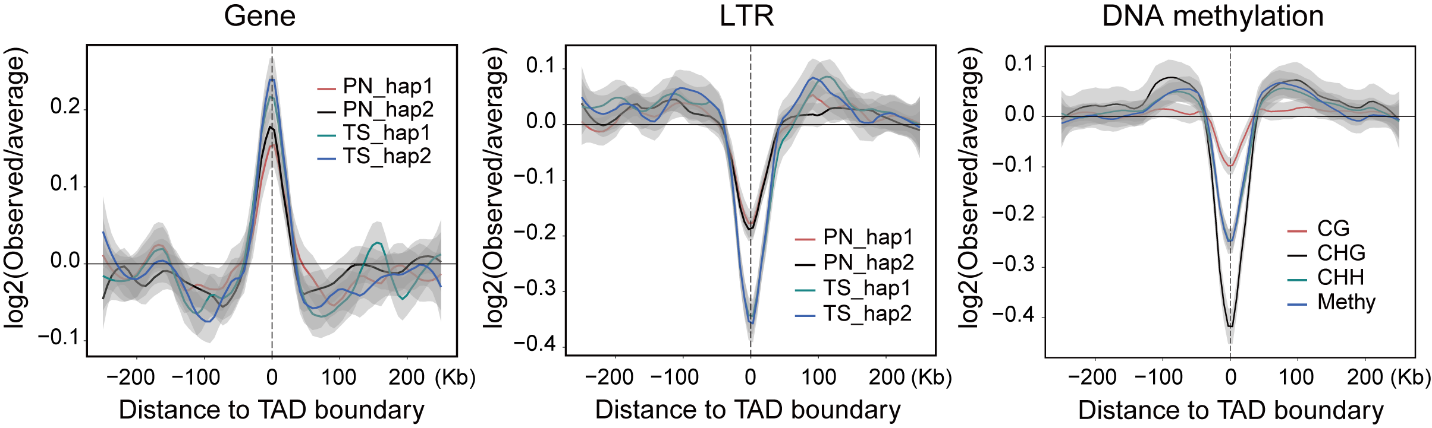
**

**Figure S10. Aggregate profiles of genomic and epigenetic features at structural domain boundaries.** Metaplots showing the average distribution of various genomic features (e.g., gene density, LTR retrotransposon density) and DNA methylation levels (CG, CHG, and CHH contexts) centered on structural domain boundaries and their flanking regions (e.g., ± 200 kb).

**
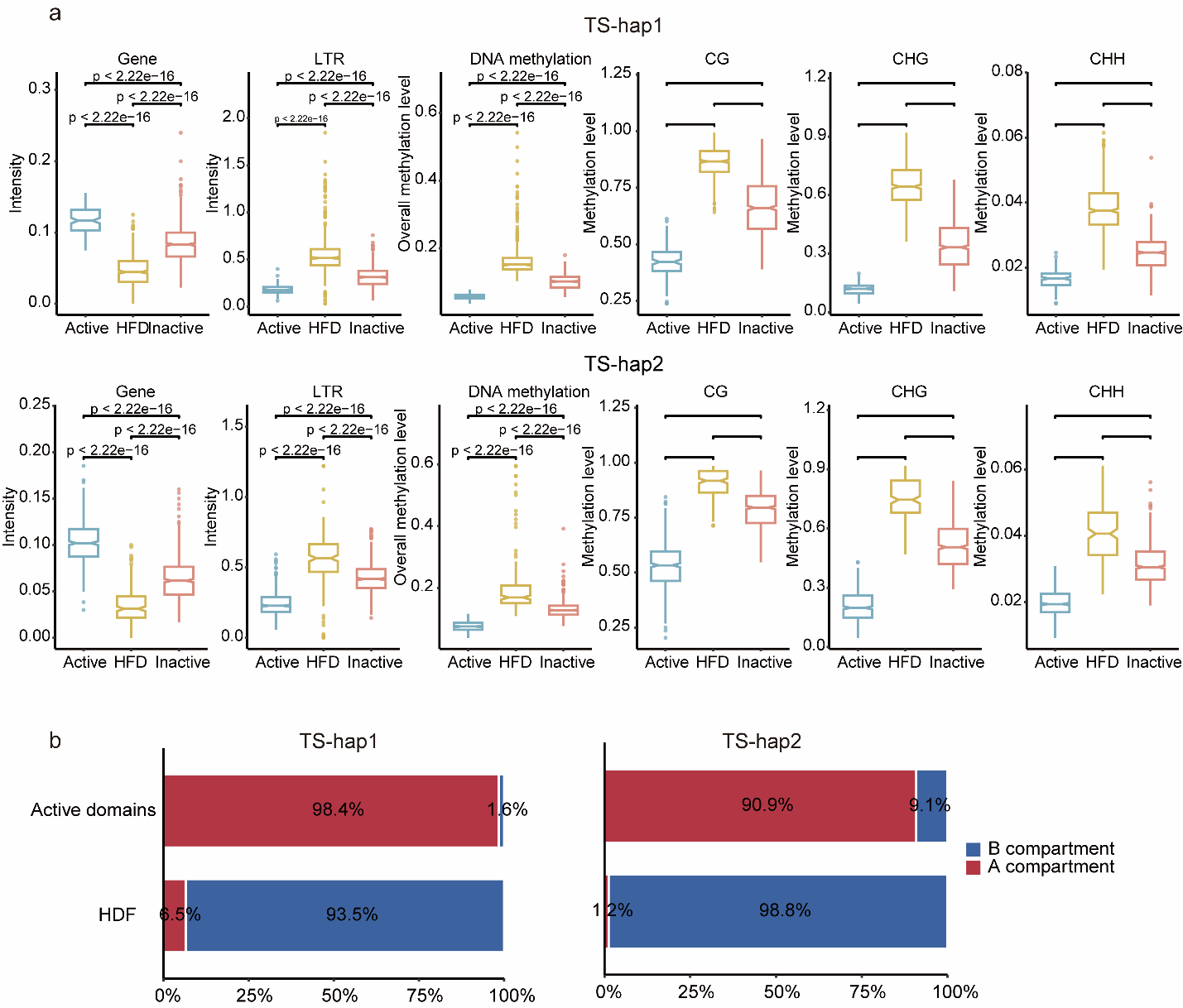
**

**Figure S11. Classification and epigenetic characterization of TAD-like domains in the TS cultivar.** (a) Comparison of genomic and epigenomic features among three distinct categories of TAD-like domains. Boxplots illustrate significant enrichment or depletion of features including gene, LTR and DNA methylation. P-values were calculated using a two-sided Wilcoxon rank-sum test. (b) Proportional overlap between the identified TAD groups and A/B compartments.

**
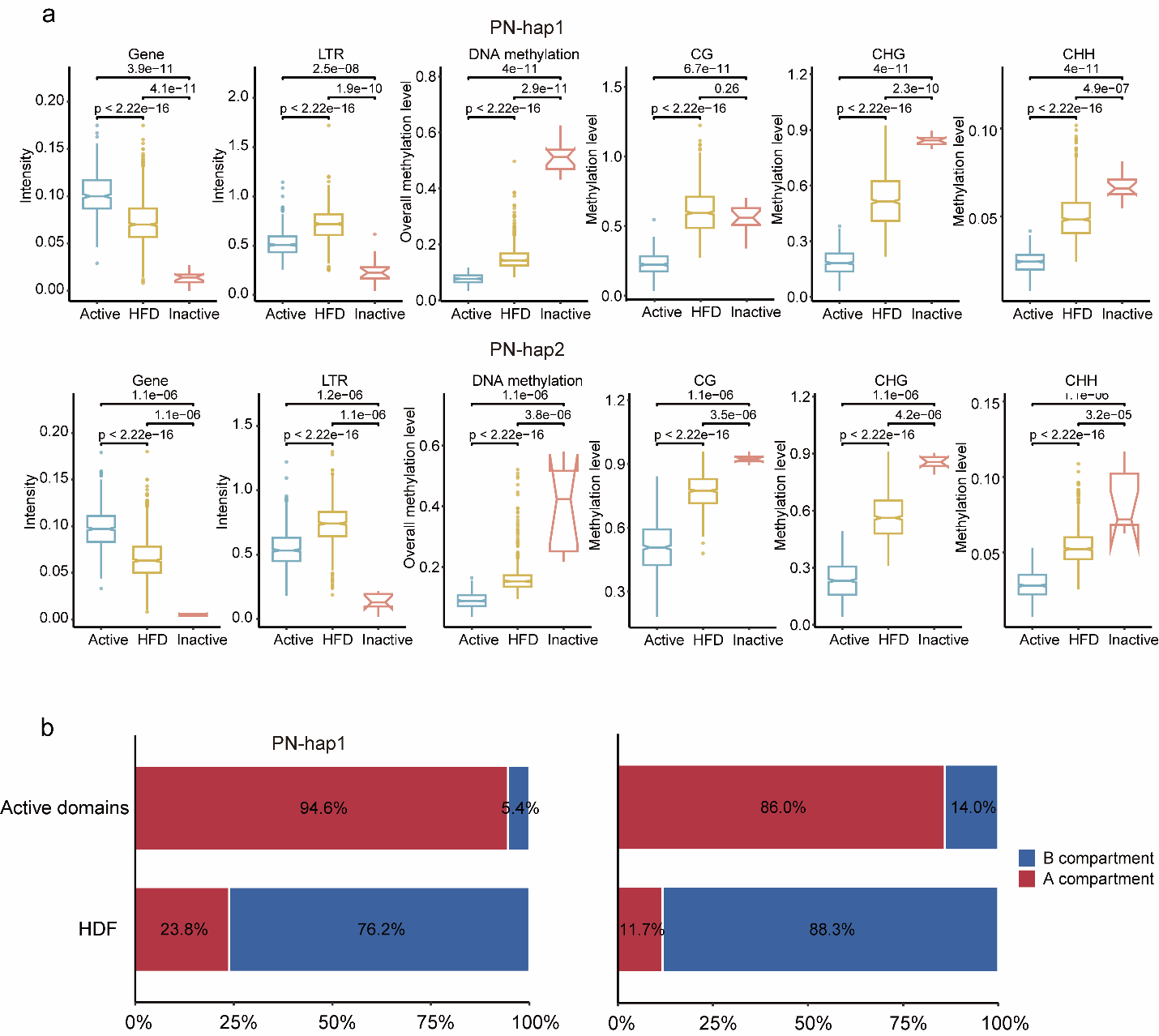
**

**Figure S12. Classification and epigenetic characterization of TAD-like domains in the PN cultivar.** (a) Comparison of genomic and epigenomic features among three distinct categories of TAD-like domains. Boxplots illustrate significant enrichment or depletion of features including gene, LTR and DNA methylation. *P*-values were calculated using a two-sided Wilcoxon rank-sum test. (b) Proportional overlap between the identified TAD groups and A/B compartments.


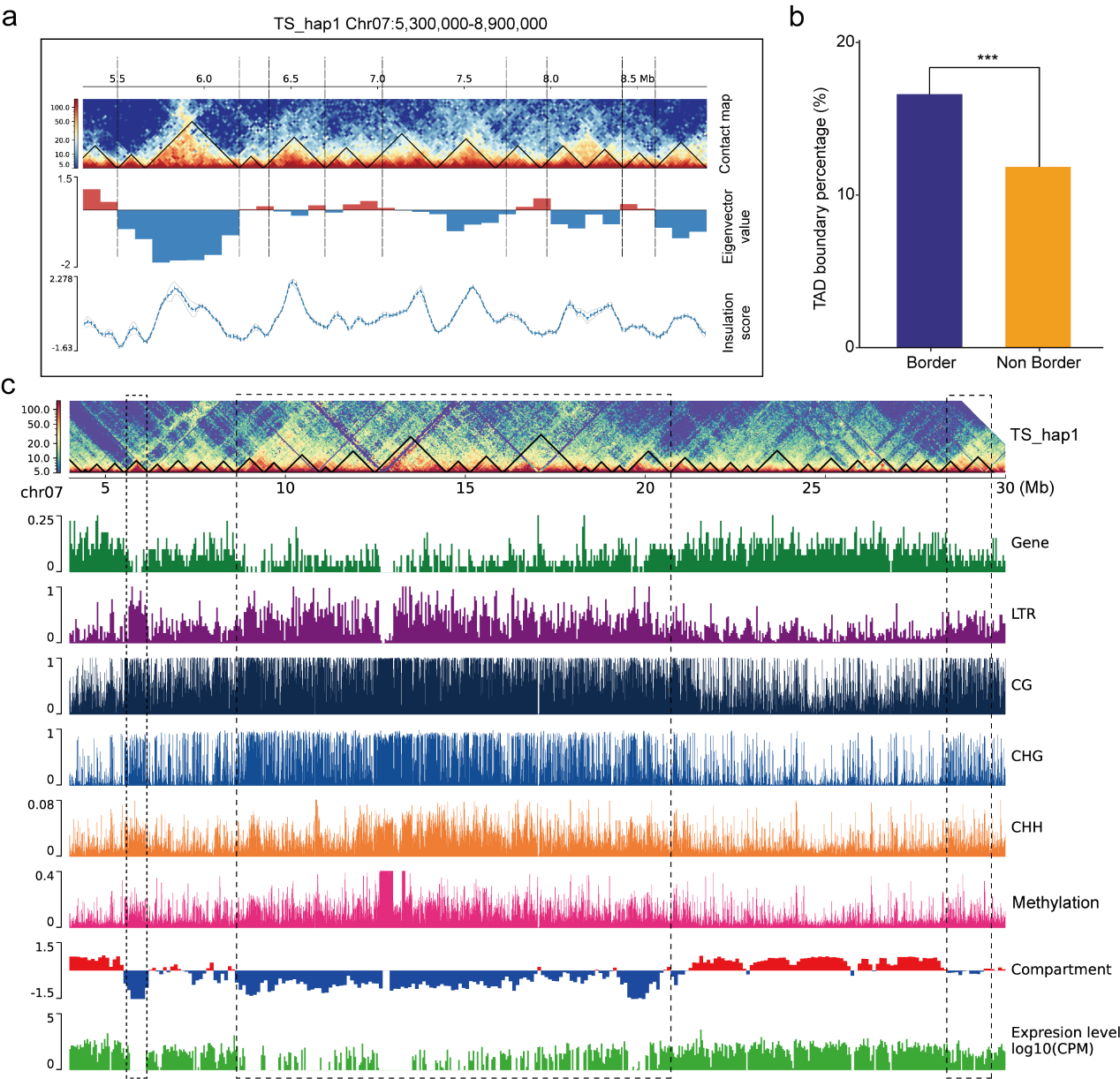


**Figure S13. Epigenetic and TE dynamics around chromatin structure.** (a) Hi-C contact map, eigenvectors, and insulation scores for the 5.3~8.9 Mb region of TS_hap1 chromosome 7. Black solid triangles indicate TADs, and black dashed lines indicate TAD boundaries. (b) Percentage of overlap between TAD boundaries and compartment boundaries and non-compartment boundaries. *** represents significant differences calculated by two-sided Fisher's exact test (*P* < 0.001). (c) Example display of the TADs in the 28 Mb region on chromosome 7. The following figure shows genomic and epigenomic characterization profiles.

**
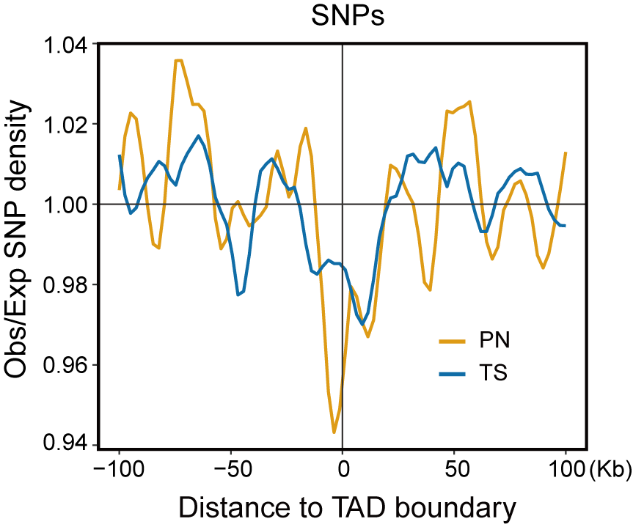
**

**Figure S14. Genomic distribution of SNPs relative to TAD boundaries.** Comparison between the observed (Obs) and expected (Exp) density of single nucleotide polymorphisms (SNPs) within a ±100 kb window flanking TAD boundaries. The expected distribution was generated based on a genome-wide background using 1,000 random permutations of boundary positions. Profiles showing the spatial enrichment/depletion of SNPs at TAD boundaries across the whole genome.

**
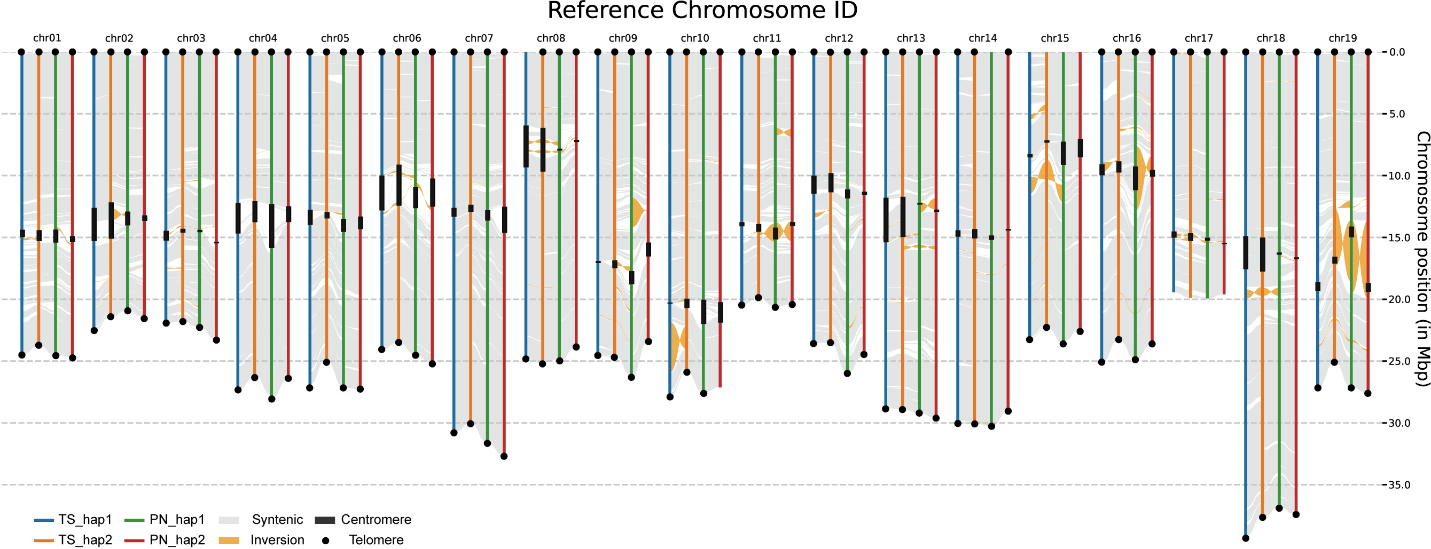
**

**Figure S15. Chromosomal synteny and large-scale inversions among four haplotype genomes.** The synteny diagram illustrates a high degree of collinearity across the four haplotype genomes of two grapevine cultivars PN and TS. Gray ribbons indicate conserved syntenic blocks, while yellow segments highlight identified large-scale chromosomal inversions. Black vertical bars denote putative centromeric regions, and terminal dots represent telomeric repeats at the chromosome ends.


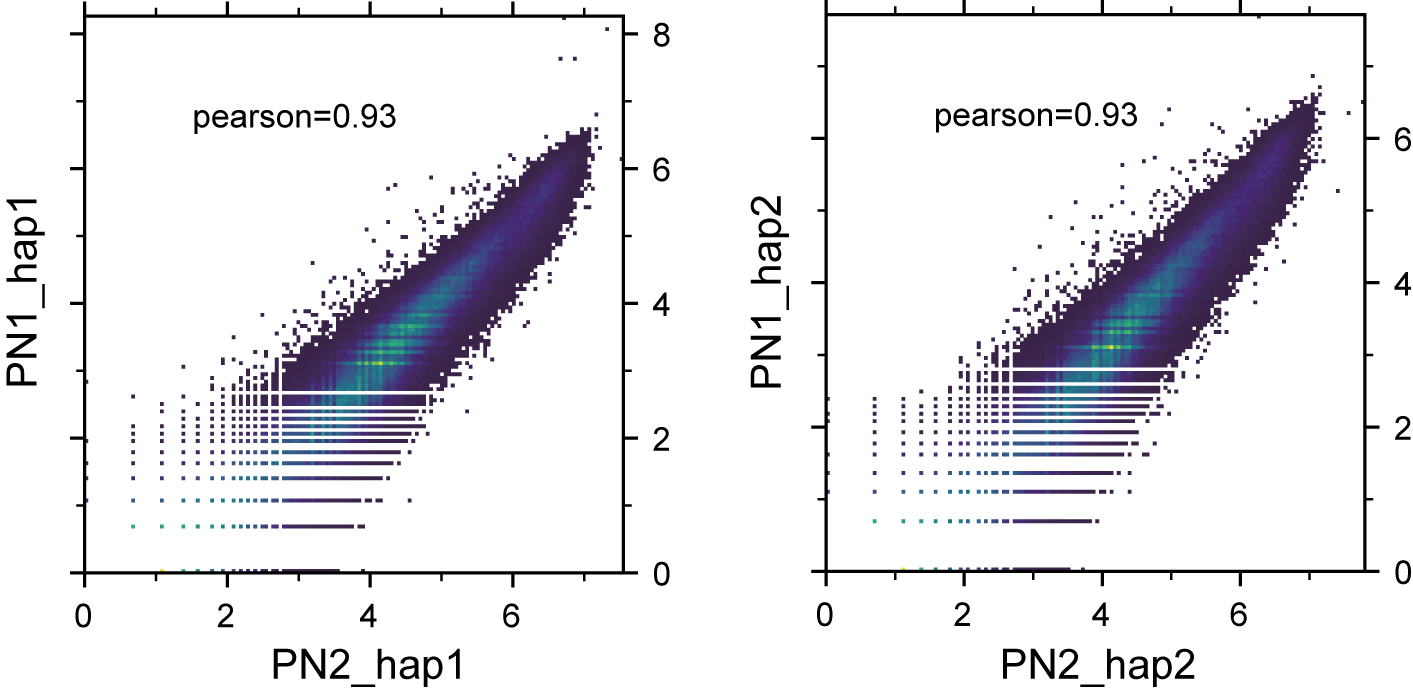


**Figure S16. Reproducibility of Hi-C biological replicates.** Scatterplot analysis showing the correlation between two biological replicates of the PN Hi-C library. The analysis was performed on 20 kb resolution contact matrices. Contact frequencies were log1p-transformed, and only interactions ranging from 5 kb to 200 kb were considered. The overall Pearson correlation coefficient is 0.93, indicating high reproducibility of the Hi-C data.

**
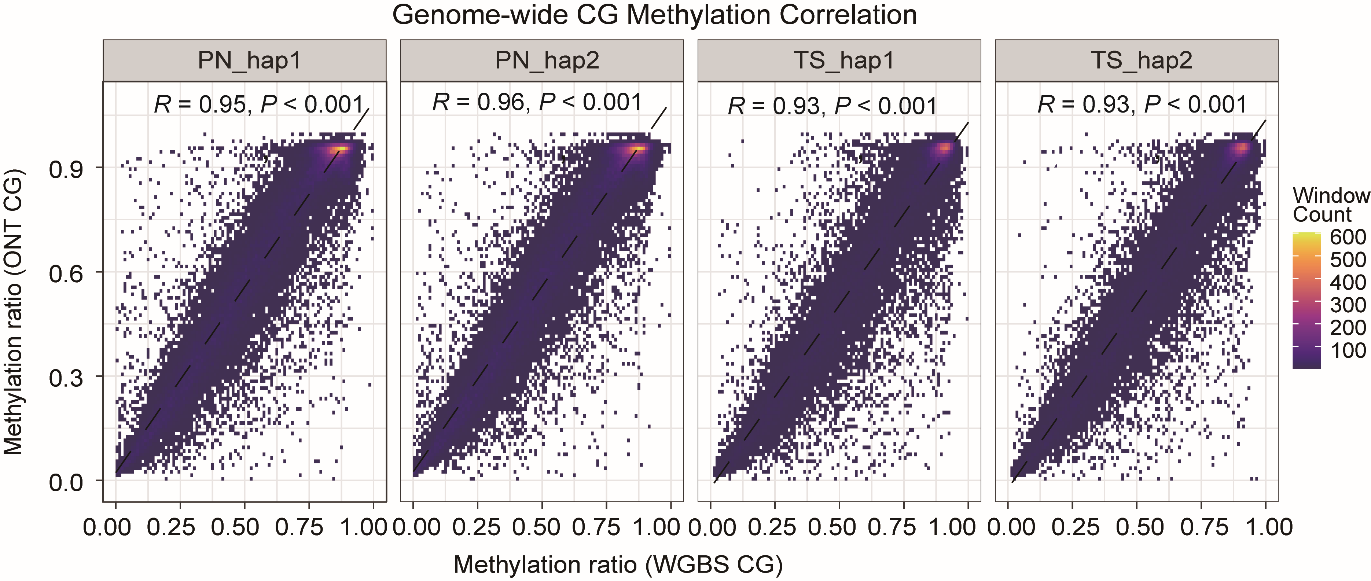
**

**Figure S17. Genome-wide concordance of CG methylation levels between WGBS and ONT sequencing platforms (R9.4.1 flow cells).** Two-dimensional density scatter plots illustrating the correlation of genome-wide CG methylation ratios derived from Whole-Genome Bisulfite Sequencing (WGBS, x-axis) and Oxford Nanopore Technologies (ONT, y-axis) across the four haplotypes (PN-hap1, PN-hap2, TS-hap1, and TS-hap2). Average methylation ratios were calculated in 4-kb genomic windows. The color gradient indicates the density of windows (Window Count) within each region, with brighter colors (yellow/orange) representing higher densities. The dashed black lines represent the linear regression fit. Pearson correlation coefficients (R) and associated *P*-values are annotated at the top of each panel, demonstrating robust cross-platform consistency ( *R* ≥ 0.93 for all haplotypes).

Table S1. Hi-C library statistics with data processed using HiCExplorer.

|  | TS_hap1 | TS_hap2 | PN_hap1 | PN_hap2 |
| --- | --- | --- | --- | --- |
| Sequenced read pairs | 198460944 | 198460944 | 279862216 | 279862216 |
| Pairs mappable, unique and high quality | 114846292(57.87%) | 115586525(58.24%) | 169005075(60.39%) | 168859896(60.34%) |
| Valid read pairs (Hi-C Contacts) | 70603360(35.58%) | 71004245(35.78%) | 117563943(42.01%) | 117468895(41.97%) |
| Inter-chromosomal | 22633465(32.06%) | 22919217(32.28%) | 34887425(29.68%) | 34771815(29.60%) |
| Intra-chromosomal | 47969895(67.94%) | 48085028(67.72%) | 82676518(70.32%) | 82697080(70.4%) |
| Short range (<20kb) | 13477369(19.09%) | 13710671(19.31%) | 23795593(20.24%) | 23743620(20.21%) |
| Long range (>=20kb) | 34492526(48.85%) | 34374357(48.41%) | 58880925(50.08%) | 58953460(50.19%) |

Note: HiC reads were aligned to both haplotypes separately and retained cross-haplotype mapped reads.

Table S2. Hi-C library statistics with data processed using HiC-Pro**.**

|  | TS_hap1 | TS_hap2 | PN_hap1 | PN_hap2 |
| --- | --- | --- | --- | --- |
| valid_interaction | 90851303 | 91233429 | 152434351 | 152053805 |
| valid_interaction_rmdup | 77729462(86%) | 78050772(86%) | 127319664(84%) | 127012553(84%) |
| trans_interaction | 24755865(27%) | 25318245(28%) | 39178958(26%) | 38924752(26%) |
| cis_interaction | 52973597 | 52732527 | 88140706 | 88087801 |
| cis_shortRange | 14229247(16%) | 14519884(16%) | 23004620(15%) | 22918644(15%) |
| cis_longRange | 38744350(43%) | 38212643(42%) | 65136086(43%) | 65169157(43%) |

Table S3. Sensitivity analysis of haplotype-specific TAD boundary variation at varying matching resolutions.

| Boundary-matching resolution | Intra-cultivar variation (95% CI) | Inter-cultivar variation (95% CI) | Wilcoxon *P*-value |
| --- | --- | --- | --- |
| 20 kb | 20.68% – 24.26% | 21.92% – 24.40% | 0.527 |
| 30 kb | 18.53% – 23.01% | 18.74% – 21.62% | 0.72 |
| 40 kb | 16.92% – 22.26% | 16.44% – 19.56% | 0.467 |
| 50 kb | 14.89% – 21.19% | 15.63% – 19.24% | 0.807 |

Table S4. Distribution of gene body methylated (gbM), unmethylated (UM), and CHG-methylated (mCHG) genes at haplotype-specific and conserved TAD boundaries in the TS and PN cultivars.

| Type | Stat | Gene | Cultivar |
| --- | --- | --- | --- |
| common | UM | 2768 | TS |
| common | gbM | 3312 | TS |
| common | mCHG | 1393 | TS |
| specific | UM | 251 | TS |
| specific | gbM | 393 | TS |
| specific | mCHG | 117 | TS |
| common | UM | 4418 | PN |
| common | gbM | 2477 | PN |
| common | mCHG | 1180 | PN |
| specific | UM | 600 | PN |
| specific | gbM | 296 | PN |
| specific | mCHG | 110 | PN |

Table S5. Median values of seven genomic and epigenomic features—including gene and LTR densities, as well as global, CG, CHG, and CHH methylation levels—across Active, Inactive, and HDF TADs.

| Status | TAD_Count | Haplotype | Gene | LTR | Expression | Methy | CG | CHG | CHH |
| --- | --- | --- | --- | --- | --- | --- | --- | --- | --- |
| Active | 141 | TS_hap1 | 0.1173204 | 0.1814317 | 57.267882 | 0.0570542 | 0.4239387 | 0.1189549 | 0.0163734 |
| HDF | 563 | TS_hap1 | 0.0461938 | 0.5410037 | 3.3934202 | 0.1635878 | 0.8613825 | 0.6573569 | 0.0384137 |
| Inactive | 838 | TS_hap1 | 0.0843499 | 0.3160531 | 28.762607 | 0.1004734 | 0.6651558 | 0.3421115 | 0.0245002 |
| Active | 571 | TS_hap2 | 0.1029649 | 0.2398608 | 43.72058 | 0.0758517 | 0.5343653 | 0.2044548 | 0.0196237 |
| HDF | 194 | TS_hap2 | 0.0343081 | 0.5522505 | 0.3531753 | 0.1966282 | 0.905819 | 0.7449282 | 0.0411017 |
| Inactive | 751 | TS_hap2 | 0.0628274 | 0.42348 | 12.437292 | 0.1302844 | 0.7857391 | 0.5111762 | 0.0312416 |
| Active | 514 | PN_hap1 | 0.1021109 | 0.5215 | 228.86966 | 0.0759975 | 0.228416 | 0.1852818 | 0.0239841 |
| HDF | 1000 | PN_hap1 | 0.072541 | 0.714228 | 55.237229 | 0.1490459 | 0.6025666 | 0.5230878 | 0.0500984 |
| Inactive | 15 | PN_hap1 | 0.0132667 | 0.249 | 1.1804547 | 0.5105533 | 0.552586 | 0.8430187 | 0.0668573 |
| Active | 723 | PN_hap2 | 0.0976888 | 0.5446376 | 33.047628 | 0.0885694 | 0.5069696 | 0.2355601 | 0.0285078 |
| HDF | 774 | PN_hap2 | 0.0653979 | 0.7353902 | 5.6286388 | 0.162085 | 0.7706038 | 0.5733516 | 0.0536185 |
| Inactive | 8 | PN_hap2 | 0.00475 | 0.13325 | 0.0389215 | 0.3964479 | 0.9250635 | 0.8534436 | 0.0831145 |

Table S6. Summary of homologous gene pairs and differentially expressed genes (DEGs) in TS and PN cultivars.

| Category | TS | PN |
| --- | --- | --- |
| Homologous gene pairs in TADs | 14970 | 15477 |
| DEGs in TADs | 199 | 213 |
| Up-regulated | 114 | 118 |
| -Down-regulated | 85 | 95 |
| Non-DEGs in TADs | 14771 | 15264 |

Table S7. Association between haplotype-specific TAD state transitions and differential gene expression in TS and PN cultivars.

| TAD_shift_type | Total | Down_Count | Down_Percent | Cultivar |
| --- | --- | --- | --- | --- |
| Active->Active | 8982 | 39 | 0.00434 | PN |
| Active->Inactive | 146 | 4 | 0.0274 | PN |
| Inactive->Active | 2534 | 22 | 0.00868 | PN |
| Inactive->Inactive | 3308 | 35 | 0.0106 | PN |
| Active->Active | 3316 | 11 | 0.00332 | TS |
| Active->Inactive | 39 | 3 | 0.0769 | TS |
| Inactive->Active | 6952 | 48 | 0.0069 | TS |
| Inactive->Inactive | 5170 | 52 | 0.0101 | TS |

Table S8. MYBA homolog genes in the phased genomes.

| PN_hap1 (7) | PN_hap2 (8) | TS_hap1 (2) | TS_hap2 (5) |
| --- | --- | --- | --- |
| *PNT2TA02G001223* | *PNT2TB02G001220* | *Vitis02g01144* | *Vitis02g01104* |
| *PNT2TA02G001231* | *PNT2TB02G001228* | *Vitis02g01151* | *Vitis02g01110* |
| *PNT2TA02G001232* | *PNT2TB02G001246* |  | *Vitis02g01111* |
| *PNT2TA02G001233* | *PNT2TB02G001229* |  | *Vitis02g01113* |
| *PNT2TA02G001242* | *PNT2TB02G001240* |  | *Vitis02g01114* |
| *PNT2TA02G001235* | *PNT2TB02G001232* |  |  |
| *PNT2TA02G001243* | *PNT2TB02G001245* |  |  |
|  | *PNT2TB02G001247* |  |  |
